# Macrophage migration inhibitory factor blockade reprograms macrophages and disrupts prosurvival signaling in acute myeloid leukemia

**DOI:** 10.1038/s41420-024-01924-5

**Published:** 2024-03-28

**Authors:** Caroline Spertini, Alexandre P. Bénéchet, Flora Birch, Axel Bellotti, Mónica Román-Trufero, Caroline Arber, Holger W. Auner, Robert A. Mitchell, Olivier Spertini, Tatiana Smirnova

**Affiliations:** 1 Service and Central Laboratory of Hematology, Lausanne University Hospital (CHUV), 1011 Lausanne, Switzerland; 2 In Vivo Imaging Facility (IVIF), Department of Research and Training, Lausanne University Hospital and University of Lausanne, Lausanne, 1011 Switzerland; 3 Department of oncology UNIL-CHUV, Lausanne University Hospital (CHUV), University of Lausanne (UNIL), 1011 Lausanne, Switzerland; 4 Ludwig Institute for Cancer Research Lausanne, 1015 Lausanne, Switzerland; 5 Faculty of Biology and Medicine, University of Lausanne, 1011 Lausanne, Switzerland; 6 Service of Immuno-oncology, Lausanne University Hospital (CHUV), 1011 Lausanne, Switzerland; 7 Department of Surgery, Division of Immunotherapy, University of Louisville, Louisville, KY 40202 USA

**Keywords:** Acute myeloid leukaemia, Cancer microenvironment

## Abstract

The malignant microenvironment plays a major role in the development of resistance to therapies and the occurrence of relapses in acute myeloid leukemia (AML). We previously showed that interactions of AML blasts with bone marrow macrophages (MΦ) shift their polarization towards a protumoral (M2-like) phenotype, promoting drug resistance; we demonstrated that inhibiting the colony-stimulating factor-1 receptor (CSF1R) repolarizes MΦ towards an antitumoral (M1-like) phenotype and that other factors may be involved. We investigated here macrophage migration inhibitory factor (MIF) as a target in AML blast survival and protumoral interactions with MΦ. We show that pharmacologically inhibiting MIF secreted by AML blasts results in their apoptosis. However, this effect is abrogated when blasts are co-cultured in close contact with M2-like MΦ. We next demonstrate that pharmacological inhibition of MIF secreted by MΦ, in the presence of granulocyte macrophage-colony stimulating factor (GM-CSF), efficiently reprograms MΦ to an M1-like phenotype that triggers apoptosis of interacting blasts. Furthermore, contact with reprogrammed MΦ relieves blast resistance to venetoclax and midostaurin acquired in contact with CD163^+^ protumoral MΦ. Using intravital imaging in mice, we also show that treatment with MIF inhibitor 4-IPP and GM-CSF profoundly affects the tumor microenvironment in vivo*:* it strikingly inhibits tumor vasculature, reduces protumoral MΦ, and slows down leukemia progression. Thus, our data demonstrate that MIF plays a crucial role in AML MΦ M2-like protumoral phenotype that can be reversed by inhibiting its activity and suggest the therapeutic targeting of MIF as an avenue towards improved AML treatment outcomes.

## Introduction

Acute myeloid leukemias (AML) are aggressive hematological malignancies with poor long-term prognoses [1]. They are complex diseases with high biological, genetic, and epigenetic heterogeneity and variable responses to treatment [2]. In the absence of stem cell transplantation, most current therapies fail to cure intermediate and high-risk AML, leading to the persistence of resistant leukemia subclones and disease relapse [3, 4]. The crucial role of allogeneic transplantation in achieving better treatment outcomes suggests that immune defense plays a central role in the disease. Additionally, both the protumoral orientation of macrophages (MΦ) in the tumor microenvironment (TME) and the expression of the “don’t eat me” signal induced by the interactions of CD47 on AML blasts with signal regulatory protein α on MΦ, contribute to promoting resistance to treatment. These observations suggest that the TME plays a central role in AML cell escape from treatment (reviewed in [5]).

Macrophage migration inhibitory factor (MIF) is a pleiotropic factor with a central role in immune responses [6]. MIF is highly expressed in AML in blasts [7], and its levels are high in the plasma of AML patients compared to healthy controls [8]; it may be a novel prognosis factor in AML [9]. MIF signals to target cells via CD74/CD44 complexes, CXCR4, CXCR2, and CXCR7 [10] and possibly other receptors [11]. MIF may therefore have direct and/or indirect effects on AML blast interactions with the TME [12], on MΦ recruitment into the bone marrow (BM) [13], and on angiogenesis [14]. MIF is secreted by many cell types and is a mediator of both autocrine and paracrine survival signaling (reviewed in ref. [15]). MΦ could be an important source of MIF [16] in the TME besides AML blasts [7] and other cellular components. Whether MIF contributes to promoting AML cell survival and resistance to treatment has not been established yet.

The concept of targeting protumoral MΦ in the TME and reprogramming their phenotype as part of cancer therapy approaches has been underscored by numerous reports in solid tumors and other hematological malignancies [17–19]. CD163 expression in AML patient BM cells correlates with poor prognosis [20]. We previously showed that CD163^+^ protumoral MΦ is predominantly present in the BM of patients with AML at diagnosis [21]. We and others demonstrated the potential of MΦ repolarization by targeting the colony-stimulating factor-1 receptor (CSF1R), the principal regulator of MΦ growth, differentiation, and polarization [18, 19, 21–23]. Interestingly, similar effects were obtained in multiple myeloma (MM) by targeting MIF [18], suggesting that it contributes to MΦ polarization. The protumoral effects exerted by MIF were further supported in vivo, where MIF deficiency, or its inhibition with 4-iodo-6-phenylpyrimidine (4-IPP) [24], causes tumor-associated MΦ to revert towards an M1-like, inflammatory phenotype [18, 25, 26]. In the context of AML, whether combining MIF inhibitor with proinflammatory agonists, such as granulocyte-macrophage colony-stimulating factor (GM-CSF), which was demonstrated to be very effective in combination with CSF1R inhibition [18, 21], reprograms MΦ to an antitumoral phenotype, was not determined. Importantly, GM-CSF was found to increase the expression of inflammatory M1-like genes in combos with both CSF1R and MIF inhibitors in MM [18].

Therefore, we investigated whether pharmacological inhibition of MIF signaling reduces AML cell survival and skews MΦ function. We show that inhibiting MIF induces the apoptosis of several AML cell lines and primary patient blasts. Using a MIF inhibitor and inflammatory factor GM-CSF, we successfully reprogram protumoral MΦ derived from peripheral blood of healthy donors (HD) and from autologous BM of AML patients into antitumoral/inflammatory M1-like MΦ. Importantly, we demonstrate that contact with fully reprogrammed MΦ promotes apoptosis of human AML cell lines and primary AML blasts in BM co-cultures. Moreover, MΦ reprogramming overcomes AML blast resistance to two standard anti-AML therapies, the FMS-like tyrosine kinase 3 (FLT3) inhibitor midostaurin and the *B-cell lymphoma 2* (BCL-2) inhibitor venetoclax. As MIF also promotes the pro-angiogenic activity of monocytes [14], we hypothesized that angiogenesis may be impaired by MIF inhibition in the AML microenvironment. Indeed, using intravital imaging, we show that MIF inhibition combined with GM-CSF administration in preclinical models reduces leukemia burden and results in a striking inhibition of angiogenesis in the TME in vivo, accompanied by a shift in MΦ phenotype.

## Results

### MIF inhibition induces AML blast apoptosis, but BM cells are protective

We first confirmed MIF secretion by primary human AML blasts from two different patients, from HL-60 and U937 AML cell lines and from HD MΦ stimulated with macrophage colony-stimulating factor (M-CSF, M-MΦ) or treated with GM-CSF and 4-IPP (R^M►GM/IPP^-MΦ) by measuring MIF in the culture supernatant with semi-quantitative cytokine arrays (Fig. S1A). Next, to determine if autocrine and/or paracrine MIF signaling may be involved in our experimental models, we analyzed by FACS the expression of MIF receptors CD74, CD44 and CXCR4 on primary AML blasts (Fig. S1B) and MΦ (Fig. S1C) from newly diagnosed patients, as well as on M-MΦ (activated by M-CSF), on CM-MΦ (activated by AML conditioned medium, CM), and on reprogrammed MΦ (R-MΦ) (Fig. S1D). Both CD74 and CD44 were consistently high on AML blasts and MΦ, whereas CXCR4 expression was heterogeneous on both cell types (Fig. S1B–G). AML cell lines express consistently CD74, CD44, and CXCR4 [27–29]. The expression of CXCR2, a non-cognate receptor for MIF [10], was analyzed in a few samples and was not consistently detectable on either blasts or MΦ.

**Fig. 1:**
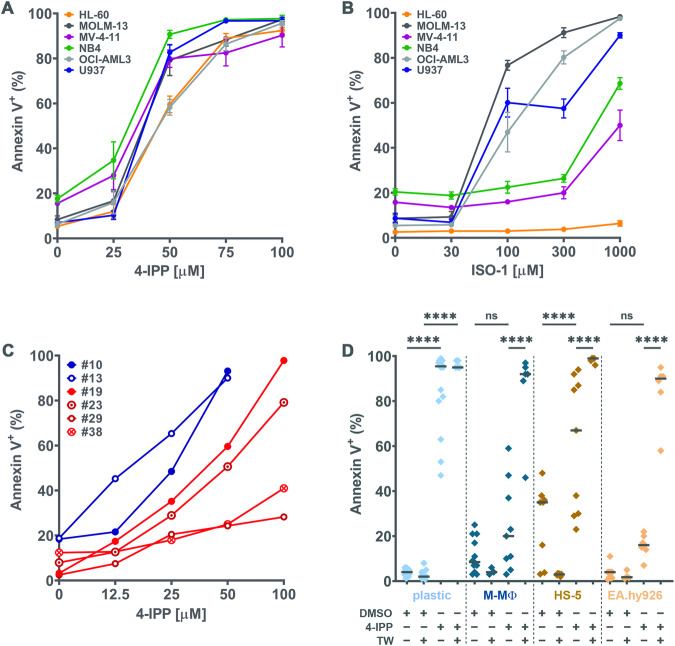
MIF inhibitors induce blast apoptosis, but BM cells are protective. **A** and **B** The indicated leukemia cell lines were cultured for 72 h with increasing doses of **A** 4-iodo-6-phenylpyrimidine (4-IPP) or **B** ISO-1 and then analyzed for apoptosis by flow cytometry staining with Annexin V. Mean ± SEM of at least three independent experiments is illustrated. **C** Each curve represents one experiment with blasts from 6 acute myeloid leukemia patients with high (#19, 23, 29, 38; red curves) or intermediate (#10, 13; blue curves) genetic risk was exposed to increasing concentrations of 4-IPP for 4–7 days. Blast cell death was identified by Annexin V staining and analyzed by flow cytometry. **D** U937 cell line was cultured in plastic wells or co-cultured on M-MΦ or HS-5 or EA.hy926 monolayers ±50 μM 4-IPP (or vehicle = DMSO) ± Transwells (TW) for 72 h, after which apoptosis was detected with Annexin V staining. The median is represented by a horizontal line. *n* = 3–23 technical repeats from *n* ≥ 3 biological repeats. ****p < 0.0001.

Since MIF is secreted by blasts which also express its main receptors, we investigated whether blast proliferation or viability was dependent on MIF. For this, cell lines representing several types of AML were incubated with increasing doses of two MIF inhibitors. MOLM-13, OCI-AML3, and U937 cells that had been stained with PKH26 were incubated with either 50 μM 4-IPP, a specific suicide substrate inhibitor [24] (Fig. S2A), or 300 μM ISO-1, another well-characterized prototypical MIF inhibitor (Fig. S2B). Cell proliferation was significantly decreased for the three cell lines and with both inhibitors by 48 h of incubation. Next, after 72 h with 4-IPP, HL-60, MOLM-13, MV-4-11, NB4, OCI-AML3 and U937 cells underwent apoptosis in a dose-dependent manner (Fig. 1A). ISO-1 induced apoptosis efficiently in MOLM-13, OCI-AML3 and U937 leukemia cell lines, but at higher concentrations than 4-IPP (Fig. 1B), which was expected as for proliferation [24]. Malignant blasts from 6 AML patients were also cultured for 4–7 days in PM containing DMSO or increasing doses of 4-IPP, which induced their apoptosis (Fig. 1C; see Table S1 for patient characteristics). Finally, apoptosis of U937, NB4, and OCI-AML3 cells induced by MIF inhibition was significantly decreased by the pan-caspase inhibitor Z-VAD-FMK (Fig. S2C, D). These results demonstrate the importance of MIF signaling to the survival and proliferation of AML cells.

Since stromal [7] and endothelial [30] cells, present in the BM, have previously been described to confer resistance to therapy, U937 cells were cultured on M-MΦ, fibroblasts (HS-5) or endothelial cell (EA.hy926) monolayers (Fig. 1D). All three cell types efficiently protected U937 from 4-IPP-induced apoptosis by direct contact, compared to plastic culture wells; this protection dramatically decreased when U937 cells were separated from monolayers by Transwell (TW) inserts in the presence of 4-IPP, but not DMSO control. Thus, in the context of a malignant microenvironment, short-term simple inhibition of MIF is unlikely to affect AML blast survival.

### MIF Inhibition with 4-IPP and exposure to GM-CSF reprograms CD163+ MΦ

**Fig. 2:**
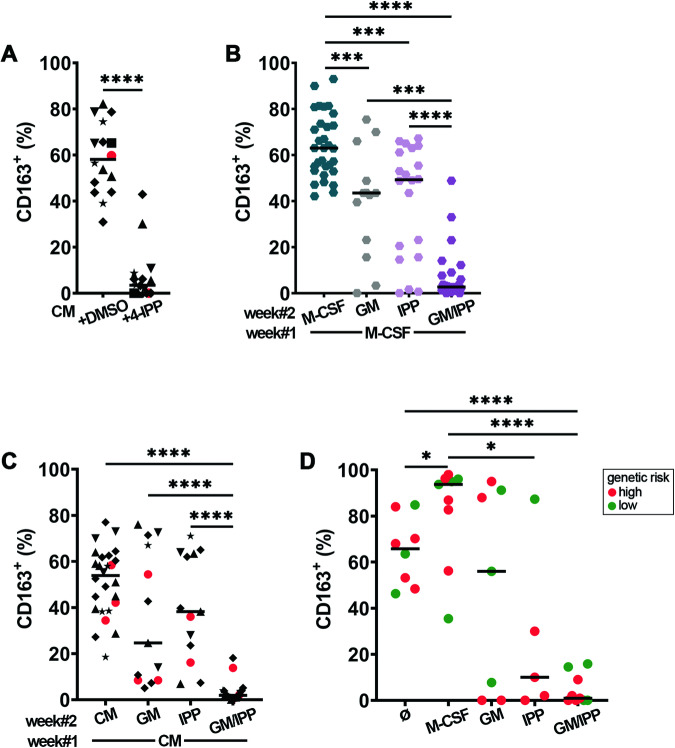
CD163 expression on macrophages is downregulated with 4-IPP and reprogramming conditions. **A** HD monocytes were cultured for 7 days in a conditioned medium from primary patient blasts (red) or leukemia cell lines (black symbols), supplemented with DMSO or 50 μM 4-IPP (IPP) before analyzing CD163 expression by flow cytometry. The conditioned medium was from ★ = HL-60, ▲ = NB4, and ♦ = U937, red dots symbolize primary acute myeloid leukemia of high genetic risk; *n* = 16. (B) Expression of CD163 on healthy donor macrophages cultured a first week in plain medium supplemented with M-CSF and for a second week in medium containing the reagents indicated on the bottom axis. Measurements from *n* = 6 different HD. **C** Same as in **B** but with CM-MΦ exposed for 1 week to blast culture medium before switching, for week#2, to the indicated reprogramming conditions. *n* = 13–26 HD. **D** Primary bone marrow patient samples containing all cell types, including blasts and macrophages, were cultured for 7 days in plain medium supplemented as indicated. Autologous macrophages were then analyzed by flow cytometry for CD163 expression. The horizontal line represents the median percentage of CD163 expression. *n* = 5–9 patient samples. *p < 0.05, ***p < 0.0005, ****p < 0.0001.

To assess whether MIF inhibition has an impact on MΦ polarization, HD monocytes were cultured with AML CM ± 4-IPP for 7 days (Fig. 2A). When cultured with AML primary blast or cell line conditioned medium (CM), monocytes differentiated into MΦ (CM-MΦ), which highly expressed the M2-like marker CD163 [21], whereas simultaneously inhibiting MIF with 4-IPP drastically impaired or even abolished CD163 expression. This highlights the importance of MIF, amongst other secreted AML factors, in protumoral MΦ activation.

To test whether established protumoral MΦ polarization is skewed by MIF inhibition, M-CSF supplementation was switched after 1 week to 4-IPP or inflammatory stimulus GM-CSF [21] or the combination of both for another week (Fig. 2B). Exposure to GM-CSF or 4-IPP alone variably lowered CD163 expression on HD MΦ compared to those cultured with M-CSF. When GM-CSF was combined with 4-IPP, the expression of CD163 on the MΦ surface was efficiently reduced or even abolished. ISO-1 was not efficient in lowering CD163 expression on M-MΦ either alone or in combination with GM-CSF (Fig. S3). We therefore used 4-IPP for all following testing. Similarly, reprogramming CM-MΦ with single molecules was less efficient than combining 4-IPP + GM-CSF, which nearly abolished CD163 expression (Fig. 2C). Finally, FC analysis of AML patients BM samples cultured for 7 days in PM (Ø) showed that most MΦ within the co-cultures expressed high levels of CD163, which was further increased when they were cultured with M-CSF (Fig. 2D). Addition of 4-IPP partially but significantly lowered CD163 expression, and 4-IPP + GM-CSF had the strongest and most significant effect on inhibiting surface CD163 expression.

We also analyzed the expression of CD80, a marker typically associated with inflammation/M1-like phenotype [31]. CD80 expression significantly increased on R-MΦ upon reprogramming with GM-CSF + 4-IPP (Fig. S4A, B). CD80 was highly variable on primary MΦ from AML patient co-cultures, with the highest trending expression upon the addition of GM-CSF + 4-IPP (Fig. S4C). Thus far, our results show that MIF inhibition or GM-CSF stimulation variably repolarizes protumoral MΦ and is more efficient in combination, as evidenced by the downregulation of CD163 expression.

### Co-culture on reprogrammed macrophages induces AML blast apoptosis

**Fig. 3:**
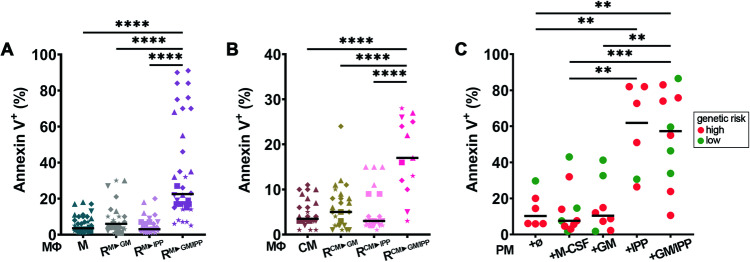
Reprogrammed MΦ and 4-IPP induce myeloblast apoptosis in co-culture experiments. **A**, **B** AML cell lines were cultured for 4 days on monolayers of M-, or R-MΦ (**A**) or monolayers of CM- or R-MΦ (**B**) and analyzed by flow cytometry for apoptosis with Annexin V staining. **A** *n* = 25–38 on 13 different HD; **B** *n* = 13–26 on 3 different HD. ★ = HL-60, ■ = MV-4-11, ▲ = NB4, ▼ = OCI-AML3, and ♦ = U937. **C** Primary myeloblast apoptosis induced after 4–7 days in indicated BM co-culture conditions. *n* = 6–10. Horizontal lines in **A**–**C** panels represent the median apoptosis frequency. **p < 0.005, ***p < 0.0005, ****p < 0.0001.

We next investigated whether AML blast survival is affected by co-culture with reprogrammed R-MΦ monolayers. MΦ that had been fully reprogrammed with 4-IPP + GM-CSF induced the death of the co-cultured AML cell lines significantly more than M- or CM-MΦ (Fig. 3A, B). Partially reprogrammed MΦ with 4-IPP or GM-CSF alone had no effect on AML apoptosis, suggesting that macrophage phenotype needs to be profoundly changed on multiple levels to affect AML blast survival. We then assessed whether the survival of primary AML blasts with different genetic mutations and risk categories, co-cultured in their own autologous BM microenvironment (cells contained in patient samples including blasts, MΦ, lymphocytes, stroma) was affected by reprogramming conditions. The continuous presence of 4-IPP significantly increased primary blast apoptosis with or without GM-CSF, regardless of genetic risk or patient macrophage proportions (ranging from 10% to 41.2%) in analyzed cocultures (Figs. 3C and S5 for normalized results according to % macrophages per sample). GM-CSF alone had no effect on primary blasts compared to PM or M-CSF. Our results thus far demonstrate that AML blast survival is directly targeted by MIF inhibition, and this is more efficient when MΦ has been reprogrammed with the MIF inhibitor 4-IPP combined with GM-CSF, reversing the pernicious effects of blasts on MΦ.

### Macrophage reprogramming with 4-IPP reverses drug resistance

**Fig. 4:**
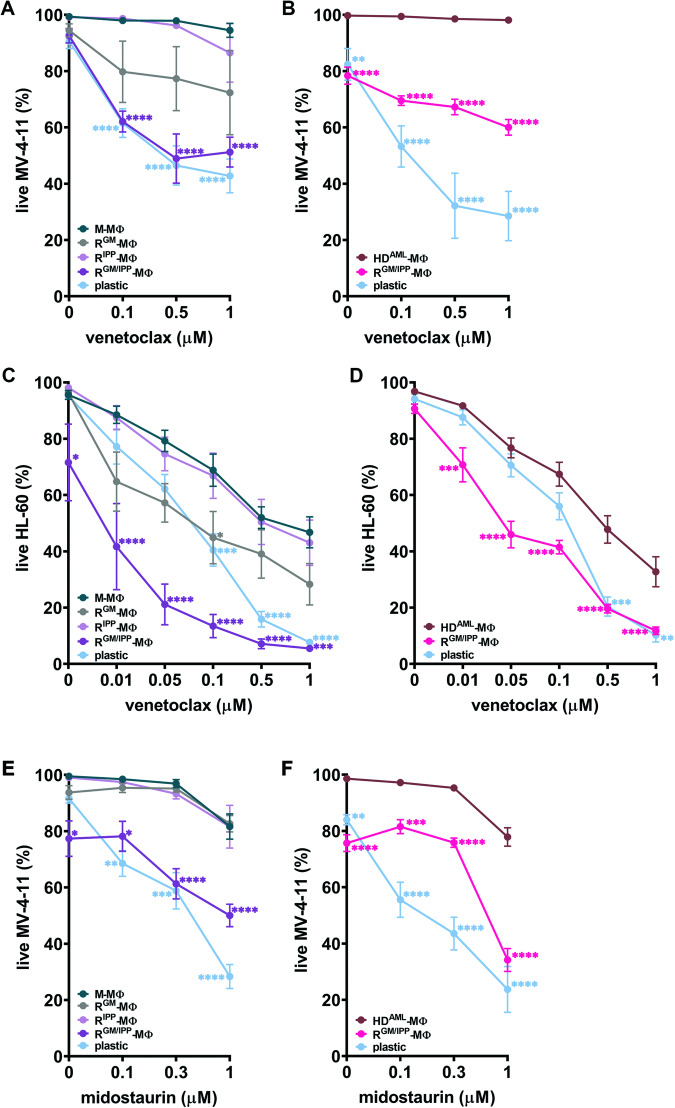
Macrophage reprogramming reverses myeloblast resistance to venetoclax and midostaurin. **A** and **B** MV-4–11 or **C**, **D** HL-60 were cultured in plastic or on indicated macrophage monolayer with increasing concentration of venetoclax for 48 h after which the percentage of live cells was measured by flow cytometry with Annexin V and/or 7-aminoactinomycin D staining. **E** and **F** MV-4-11 were cultured in plastic or on indicated macrophage monolayer with increasing concentration of midostaurin for 48 h after which the percentage of live cells was measured by flow cytometry with Annexin V and/or 7-aminoactinomycin D staining. *p < 0.05, **p < 0.005, ***p < 0.0005, ****p < 0.0001 compared to M-macrophages (**A**, **C**, **E**) or to CM-macrophages (**B**, **D**, **F**); *n* = 3–8 different HD.

Venetoclax, a BCL-2 inhibitor, and midostaurin, a FLT3 inhibitor, are both used to treat AML patients [32, 33]. MV-4-11 AML cells sensitive to anti-apoptotic BCL-2 protein inhibition on “plastic” [34], cultured directly on monolayers of M- or CM-MΦ, were resistant to up to 1 μM venetoclax (Fig. 4A, B), a phenomenon we previously demonstrated [21]. However, co-culturing them with R^M►GM/IPP^- or R^CM►GM/IPP^-MΦ effectively resensitized them to venetoclax. By contrast, singly treated R^M►IPP^-MΦ had no impact on resensitizing MV-4-11 cells to venetoclax (Fig. 4A). We next tested the sensitivity of BCL-2 inhibitor-responsive HL-60 cells [34] in the same conditions. Compared to M- or CM-MΦ, co-culture on R^M►GM/IPP^- or R^CM►GM/IPP^-MΦ restored a mortality frequency similar to or higher than that measured on plastic (Fig. 4C, D). On the other hand, HL-60 viability on R^M►IPP^-MΦ was not different from what was observed on M-MΦ (Fig. 4C). Interestingly, both cell lines are partially re-sensitized to Venetoclax on R^M►GM^-MΦ (Fig. 4A and C). R^M►GM^-MΦ, while not fully reprogrammed, may affect response to BCL-2 inhibition in leukemia cells by changing their secreted cytokine profile [21] or other mechanisms [18]. We next demonstrate that both HL-60 and MV-4-11 cell lines cultured in direct contact with M-MΦ monolayers strikingly downregulate BCL-2 to levels undetectable by immunoblotting (Fig. S7, uncropped original gel shown), as compared to plastic wells or R^M►GM/IPP^ monolayers. This delineates one way protumoral MΦ induce AML cell resistance.

The MLL-AF4 and FLT3-ITD mutated MV-4-11 cells co-cultured in direct contact with M- or CM-MΦ were also resistant to midostaurin (Fig. 4E, F) [21]. Co-culturing them with R^M►IPP^- or R^M►GM^-MΦ monolayers had no effect, but R^M►GM/IPP^- and R^CM►GM/IPP^-MΦ effectively restored the sensitivity of MV-4–11 cells to midostaurin. Taken together, our results in Fig. 4 suggest that MΦ treated with combinations of 4-IPP + GM-CSF have a reduced capacity to or no longer protect myeloblasts from targeted inhibitors.

Secreted factor profile analyses of reprogrammed R^M►GM/IPP^-MΦ CM confirm a decrease in protumoral, survival-promoting factors such as IGFBP-2 [35] and VCAM-1 [36] (Table S2, Fig. S6). As expected, the secretion of several inhibitory molecules increased (Table S2). Amongst them, IL-4 was found to inhibit AML cell growth and induce apoptosis [37]. Thus, some secreted MΦ factors change along with the MΦ phenotype and may influence AML blast survival or sensitivity to inhibitors via other mechanisms or pathways that need to be investigated.

### MIF inhibition affects the malignant microenvironment and reduces leukemia burden in vivo

**Fig. 5:**
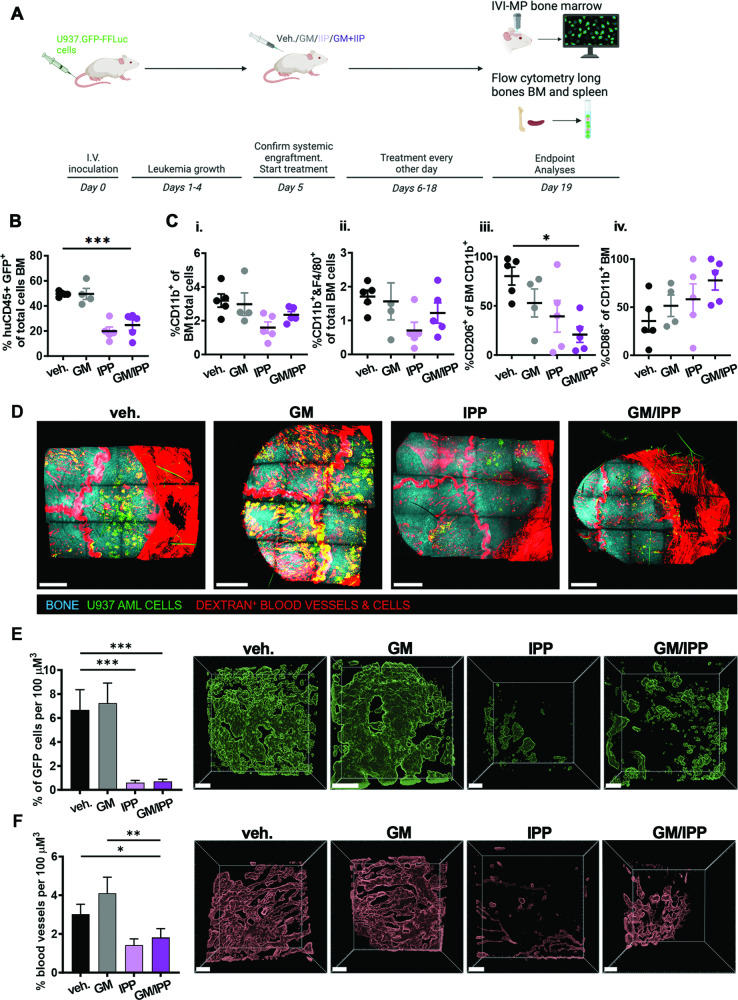
MIF inhibition significantly reduces leukemia burden in the bone marrow and affects the microenvironment in vivo. **A** Schematic representation of the experimental setup, created with BioRender. **B** Flow cytometry analyses at the end of the experiment (days 19–23 post-inoculation) of proportions of i.v. engrafted U937.GFP-FFLuc cells in bone marrow from long bones of NSG mice treated with vehicle (veh.), GM-CSF (GM, 3000 U/kg), 4-IPP (IPP, 80 mg/kg), and GM-CSF + 4-IPP (GM/IPP, administered at 3000 U/kg and 80 mg/kg body weight, respectively) starting on day 5 of engraftment. **C** Flow cytometry analysis was performed on bone marrow from femurs and tibias from mice treated as indicated for (i) % of total CD11b^+^ cells, (ii) % of CD11b^+^ and F4/80^+^ double-positive cells, (iii) % CD206^+^ M2-like macrophages, and (iv) % CD86^+^ M1-like macrophages in the CD11b^+^ population, respectively. Data are shown as the mean ± SEM (*n* = 4–5 mice per group), *p < 0.05, ***p < 0.001. **D** Representative mosaics from multiple 3-D z-stacks of whole top skull bone marrow from intravital imaging by multiphoton microscopy in mice from each treatment group show U937.GFP-FFLuc cells (green), dextran-labeled blood vessels and positive cells (red), and second harmonic generation imaging identified bone structures (cyan); scale bars = 1000 µm. **E** Quantification (mean ± SEM) of the percentage of U937.GFP-FFLuc cells per 100 µm^3^ volume of calvaria bone marrow, analyzed from 6 to 8 3-D z-stacks, *n* = 3 mice per treatment group, acquired by intravital multiphoton microscopy, using software analyses tools for surface rendering in the green channel as described in methods; to the right of the plot are representative 3-D image stacks of rendered GFP+ cells in calvaria bone marrow, scale bars = 200 µm. ***p < 0.001. **F** Quantification dextran+ vessels normalized per 100 µm^3^ bone marrow tissue volume, using 3-D surface rendering in the red channel; bars are means ± SEM (analyzed from 3 mice per group); to the right of the plot are representative 3-D image stacks of rendered dextran + vessels in calvaria bone marrow, scale bars = 200 µm. *p < 0.05, **p < 0.005.

We next tested the in vivo efficacy of 4-IPP treatments with and without murine GM-CSF in a xenograft model of leukemia using U937.GFP-FFLuc cells systemically engrafted in NSG mice. Single or double treatments were started once engraftment was confirmed on day 5 (Fig. 5A). At the end of the experiment, FC analyses of fresh bone marrow (BM) extracted from long bones showed that AML burden was significantly reduced in 4-IPP ± GM-CSF treated mice, as determined by the proportions of GFP+ humanCD45 + U937.GFP-FFLuc AML cells, while GM-CSF alone had no effect, like vehicle (Fig. 5B). U937.GFP-FFLuc cells were also engrafted in the spleens, interestingly significantly more in GM-CSF-treated mice than in both 4-IPP treatment groups (Fig. S8A). To evaluate the microenvironment along with changes in leukemia burden, FC analyses of BM show that overall proportions of murine CD11b^+^ and F4/80^+^ monocytic cells or MΦ do not change significantly upon treatments (Fig. 5Ci–ii), but the proportion of murine protumoral M2-like CD206^+^ MΦ [38] decreases significantly in double-treated mice (Fig. 5Ciii). The presence of M1-like, inflammatory CD86^+^ MΦ was slightly but not significantly increased in treated mice (Fig. 5Civ).

To directly visualize changes in the malignant microenvironment, we performed multiphoton microscopy imaging (IVI-MP) of the bone marrow in the calvaria of living mice at single-cell resolution (see Movie S1, Figs. 5D, and S8B). Image analysis software was used to create surface renderings in the green and red channels, allowing for size classification and quantification of U937.GFP-FFLuc leukemia cells and dextran-labeled blood vessels [38, 39]. Using this approach on multiple 3-D z-stacks and tumor mosaics, we demonstrated significantly reduced leukemia burden in skull BM of 4-IPP ± GM-CSF treated mice (Fig. 5E), reflecting leukemia burden in BM from long bones. Moreover, vasculature in 4-IPP-treated BM microenvironment was significantly reduced compared to vehicle or GM-CSF treatments alone (Fig. 5F).

**Fig. 6:**
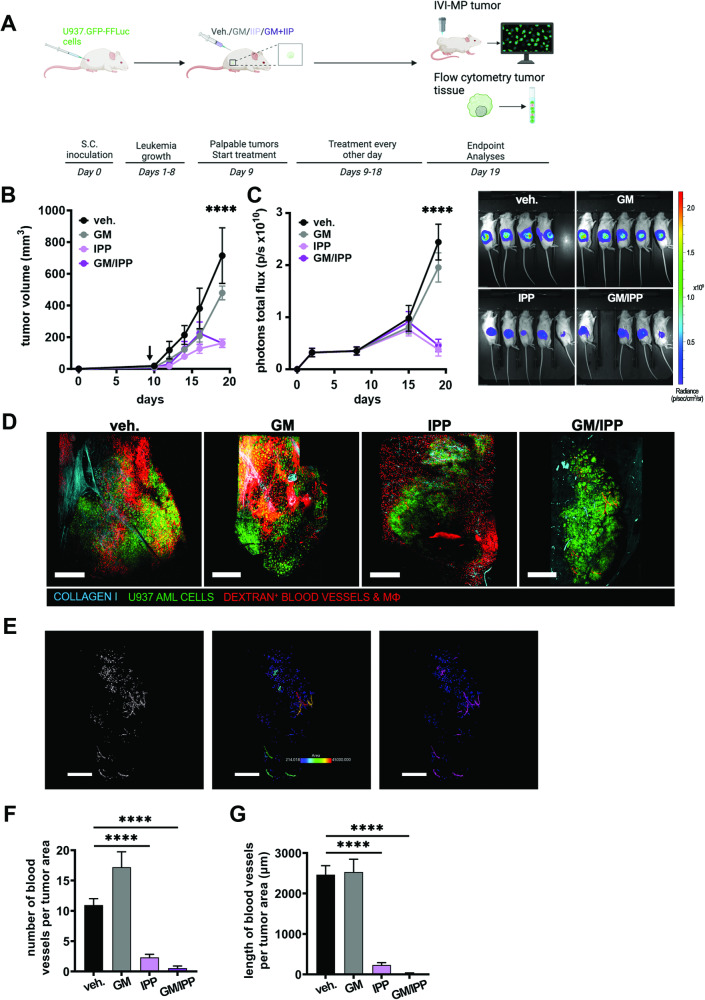
MIF inhibition attenuates extramedullary leukemia progression and affects tumor vascularization in vivo. **A** Schematic representation of the experimental setup, created with BioRender. **B**, **C** Subcutaneous U937.GFP-FFLuc leukemia cell tumor growth progression in NSG mice, day 0 (cell inoculation) until day 19 (end of the experiment); black vertical arrow indicates the start of mouse treatments with vehicle (veh.), GM-CSF (GM, 3000 U/kg), 4-IPP (IPP, 80 mg/kg), and GM-CSF + 4-IPP (GM/IPP, administered at 3000 U/kg and 80 mg/kg body weight, respectively) on day 9 of tumor growth. **A** Curves are the mean tumor volumes ± SEM (*n* = 4–5 mice per group) measured by calipers. **C** Curves are the mean total photons flux ± SEM (*n* = 4–5 mice per group) measured by bioluminescence; images show radiance at the same scale of all the tumor-bearing mice from the 4 treatment groups at the end of the experiment on day 19. ****p < 0.0001 compared to the vehicle on day 19 (**B**, **C**); *n* = 4–5 (**D**). Representative whole tumor image mosaics from intravital imaging by multiphoton microscopy of mice from each treatment group show U937-GFP leukemia cells (green), dextran-labeled blood vessels, and positive cells (red), and second harmonic generation imaging identified collagen I fibers (cyan); scale bars = 500 µm. **E** A representative illustration of image analysis approach from IVI-MP of a tumor mosaic from a GM-CSF + 4-IPP treated mouse, same tumor as in (**D**); first panel shows 3-D surface rendering in the red/dextran channel, second panel shows size classification and third (right) panel shows the separation of tumor blood vessels (purple) and phagocytic macrophages (blue); scale bars = 500 µm. **F**, **G** Quantification of the numbers (**F**) and lengths (**G**) of blood vessels normalized by tumor areas; bars are means ± SEM (images analyzed from ≥3 mice per group). ****p < 0.0001.

We next tested 4-IPP in a xenograft model of subcutaneously inoculated U937 leukemia cells, representing extramedullary disease [40]. Single or double treatments were started once tumors were formed (Fig. 6A). Bioluminescence and tumor volume measurements show that tumor progression was significantly attenuated in 4-IPP ± GM-CSF-treated mice (Fig. 6B, C).

Again, IVI-MP revealed striking differences in the TME between groups (Figs. 6D, E and S9 for representative images and Movie S2). We demonstrate that the number and length of blood vessels measured per tumor area are both significantly reduced within the 4-IPP ± GM-CSF treated tumor tissues, compared with vehicle or GM-CSF (Fig. 6F, G).

**Fig. 7:**
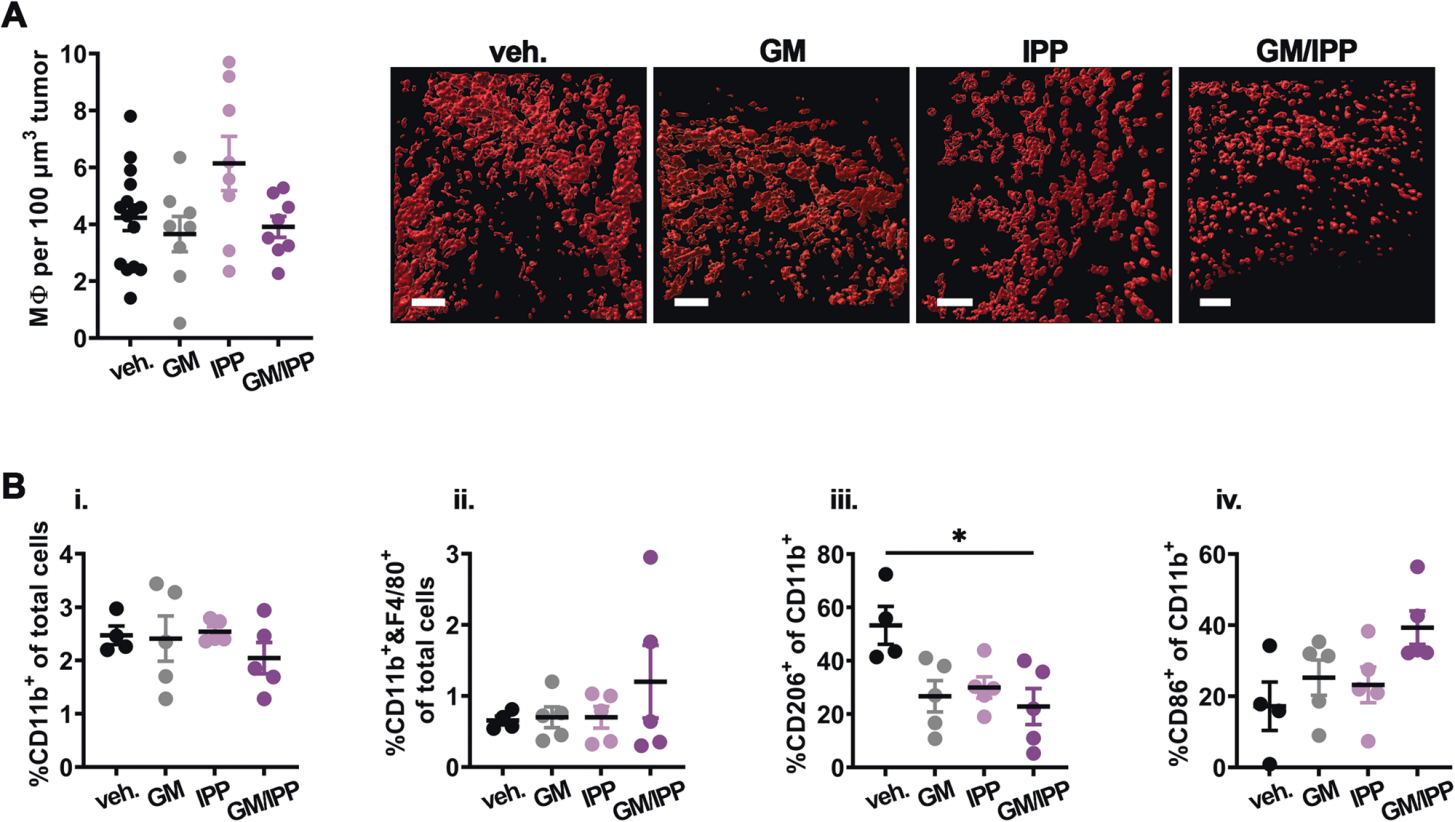
MIF inhibition in combination with GM-CSF reduces the proportion of protumoral macrophages in vivo. **A** Quantification (mean ± SEM) of numbers of dextran-positive macrophages per 100 µm^3^ of tumor tissue, analyzed from 3-D z-stacks acquired by intravital multiphoton microscopy, using software analyses tools for surface rendering and size classification as described in the “Methods” section and Fig. 6. Panels right of plot show representative examples of 3-D surface rendering in the red/dextran channel of dextran-positive macrophages classified by size from each treatment group; scale bars = 100 µm. **B** Flow cytometry analysis was performed on U937 tumors from mice treated as indicated, for (I) % of total CD11b^+^ cells, (ii) % of CD11b^+^ and F4/80^+^ double-positive cells, (iii) and % CD206^+^ M2-like macrophages and (iv) % CD86^+^ M1-like macrophages in the CD11b^+^ population, respectively. Data are shown as the mean ± SEM (*n* = 4–5 mice per group), *p < 0.05.

Further image analysis confirmed the overall presence of dextran^+^ MΦ in tumor tissues was similar in all the treatment groups (Fig. 7A). Similar to BM, the overall proportions of MΦ did not change upon treatments (Fig. 7Bi–ii), but the proportion of host protumoral M2-like CD206^+^ MΦ decreased significantly in tumor tissues from double-treated mice (Fig. 7Biii). The presence of M1-like CD86^+^ MΦ was variable (Fig. 7Biv). These results support our in vitro data and reveal additional effects on vasculature in vivo.

To further evaluate changes in the microenvironment, tumor pieces from the four groups were cultured in PM and analyzed for secreted human and mouse factors and cytokines [21, 38] using semi-quantitative arrays (Table S3). From U937 leukemia-cell secreted cytokines, 4-IPP ± GM-CSF caused a decrease in the secretion of several tumor-promoting factors, such as osteopontin [41]. Relative changes were also measured amongst murine (host) cytokines in the TME (Fig. S10, Table S4), including those involved in tumor angiogenesis [17]. MMP-9 levels were lowered by 4-IPP treatments, as were angiopoietin-2 and -3. Resistin, which reverts AML cell resistance to drugs, and AML MΦ phenotype to M1-like [42], was increased severalfold in all GM-CSF ± 4-IPP treatment groups, relative to vehicle. CCL11, an inflammatory TME cytokine [31], increased in 4-IPP + GM-CSF treated tumors. Changes in cytokines could contribute to the observed differences in tumor tissue vasculature and MΦ modulation in vivo.

## Discussion

In this work, we show that inhibiting MIF induces the death of leukemia cell lines and primary blasts and reprograms M2-like MΦ orientation when combined with GM-CSF, leading to blast cell death and reversal of resistance to therapies targeting FLT3-ITD or BCL-2 in vitro. In xenograft models, treatment with MIF inhibitor 4-IPP reduces leukemia burden, and with GM-CSF has a profound effect on the TME in vivo, notably on malignant tissue vasculature.

MIF is a pleiotropic factor that is released by multiple cell types. It is detectable in normal plasma and at much higher levels in inflammatory/infectious diseases and cancers [6]. A crucial role of MIF in promoting tumor growth was demonstrated in MIF-deficient mice [25]. One way MIF exerts its effects is by stimulating survival pathways. Protection from apoptosis may require CD44 to be an integral part of the CD74 receptor complex, which may contribute to promoting resistance to treatment by activating the Src-family kinase pathway [43]. MIF can also bind to CD74 complexed with CXCR4 or CXCR7 [10] and induce downstream MAPK and phosphoinositide 3-kinase/AKT activation. The recruitment of the hepatocyte growth factor receptor c-Met to the CD74/CD44 complex is another mechanism that promotes B-lymphocyte survival [11]. A possible mechanism of survival and resistance of AML blasts is the induction of interleukin-8 expression in BM mesenchymal stromal cells following activation of CXCR4 [7]. Interestingly, MIF secretion by FLT3 mutated AML cells increases upon tyrosine kinase inhibitor treatment, accompanied by increased CXCR2 receptor levels, resulting in blast resistance to inhibitors [44]. MIF signaling in AML blasts remains to be fully delineated and is likely influenced by TME cross-talks.

MIF is also recognized as a “pro-M2” factor and creates a protumoral microenvironment by various mechanisms [25, 45–47]. It synergizes with signals from malignant cells to polarize MΦ to an M2-like orientation [26]. In AML, we show that blasts release MIF and that MΦ cultured in AML CM exhibit an M2-like orientation that is prevented upon MIF inhibition. We also show that MIF inhibition with 4-IPP, in combination with GM-CSF, results in efficiently re-orienting the MΦ phenotype. Literature increasingly demonstrates MIF’s wider roles in immune cell regulation. 4-IPP was found to attenuate immunosuppression [46–49]. In melanoma, inhibiting MIF improves the response to immune checkpoint blockade by potentiating CD8^+^ T-cell infiltration and by reorienting MΦ polarization to a proinflammatory M1-like phenotype [45]. In MM, the M2-like orientation of MΦ is reversed to an M1-like phenotype in vitro and in vivo by inhibiting MIF in the presence of GM-CSF and MIF receptors CD74 and CXCR7 are both involved in the feedback loops promoting M2-polarization [18]. GM-CSF was found to increase the expression of inflammatory M1-like genes in combination with both CSF1R and MIF inhibitors in MM [18]. Whether these effects are similar in AML remains to be confirmed.

MIF can induce the secretion of angiogenesis-promoting factors such as VEGF [49]. The involvement of MIF in angiogenesis in different tumor types (reviewed in ref. [50]) is consistent with our quantifications of a striking effect on tumor vasculature in our in vivo model. Indeed, MIF is regulated in a HIF-dependent manner to induce new blood vessel formation. MIF blockade using antibodies were found to inhibit tumor-induced angiogenesis, and 4-IPP inhibited neoangiogenesis [50].

We show that MIF inhibition reduces leukemia cell proliferation by 24–48 h. This is in line with 4-IPP reducing proliferation and survival in other malignant cell types [51]. When 4-IPP was developed as a more potent inhibitor of MIF compared to its prototypes, its efficacy was demonstrated by strongly inhibiting lung adenocarcinoma cell migration and proliferation [24]. Consistent with this study, we also found the well-characterized biological MIF inhibitor ISO-1 to be less efficient in inhibiting AML cell survival and proliferation and to have no measurable effect on MΦ reprogramming, compared to 4-IPP. This could be due to potential differences in the biological activity of the two inhibitors. Interestingly, results from a virtual screening study indicated that while ISO-1 does inhibit MIF/CD74 binding interaction, 4-IPP does not [52]. On the other hand, 4-IPP was demonstrated to inhibit MIF secretion in monocytic cells [53] and induce proteasomal degradation of intracellular MIF, also leading to reductions in secreted MIF and subsequent autocrine and paracrine signaling (Prof. Mitchell RA, unpublished). These findings may explain why 4-IPP has stronger effects in our experiments.

Importantly, malignant cells are sensitive to 4-IPP, but not normal BM cells [54]. Our in vivo results show that 4-IPP delays leukemia progression but is not sufficient to eliminate leukemia cells in mice. Thus, it would be valuable to next explore MIF inhibition with and without GM-CSF, in combination with clinical inhibitors targeting FLT3 and BCL-2, as our in vitro experiments strongly suggest. However, GM-CSF has dual effects on blasts, which must be carefully considered. GM-CSF induces both pro- and anti-apoptotic signals and can be a stimulator of AML blast proliferation [55]. In different tumor models, GM-CSF has been shown to be either pro- or anti-tumoral, pro- or anti-angiogenic, all of which can depend on the microenvironment or directly on GM-CSF dosage [56]. Thus, future exploration on delineating the role of GM-CSF in combination with inhibitors, and fine-tuning its administration for maximum anti-tumoral effects, will be relevant.

In conclusion, our study suggests that MIF inhibition may represent a novel way to target AML blasts and their TME, overcoming resistance to inhibitors and the protective effect of protumoral MΦ towards blasts.

## Methods

### Patient samples

The experiments were done in accordance with the Declaration of Helsinki and approved by the Commission cantonale d'éthique de la recherche sur l'être humain (CER-VD protocol#2017-01509, November 9, 2017). Peripheral blood or BM was obtained from 29 AML patients ≥18 years of age diagnosed at Centre Hospitalier Universitaire Vaudois in Lausanne, Switzerland. Informed consent was obtained from all patients involved in the study. Table S1 indicates each patient’s diagnosis based on the World Health Organization 2016 classification and European LeukemiaNet 2017 risk stratification by genetics [57]. Monocytes and MΦ were obtained from HD peripheral blood and activated (7 days) as previously described to form monolayers [21] and reprogrammed using GM-CSF ± 4-IPP (7 more days), see *Supplementary Materials and Methods*. Primary patient sample analyses, co-culture assays, and AML cell death and apoptosis analyses were described previously [21] and in *Supplementary Materials and Methods*.

### Cell lines

Cell lines were described in ref. [21] and in *Supplementary Materials and Methods*.

### In vivo mouse xenograft models

Animal experimentation was performed under the authorization VD3390x1 (to C. Arber), approved by the veterinary authorities of the Canton de Vaud, Switzerland. Female NOD.Cg-*Prkdc*^scid^*IL2rg*^tm1Wjl^/SzJ NOD-SCID-γc^−/−^ (NSG) mice (6–10 weeks old) were injected intravenously (i.v.) [58] to study bone marrow/systemic leukemia burden or subcutaneously (s.c.) to represent extramedullary disease [40], with 1 × 10^6^ U937.GFP-FFLuc cells s.c. into the right flank or i.v. into tail veins. The sample size was based on similar data analyses from our previous studies [38, 59]. Systemic engraftment of U937.GFP-FFLuc cells were assessed on day 5 after i.v. tail vein injection, and s.c. tumors were all palpable by day 9. Mice for each xenograft experiment were randomly distributed into four groups after leukemia engraftment. The investigator was not blinded to the group allocation during the experiment. Treatments started on day 5 for the i.v. and day 9 for s.c. xenografts, and were administered intraperitoneally every other day in total volume of 100 μl: control vehicle (mixture of PBS, corn oil, and dimethyl sulfoxide), recombinant murine GM-CSF (3000 U/kg body weight, Cell Guidance Systems) with or without the MIF inhibitor 4-IPP (80 mg/kg body weight [25]).

### Intravital imaging by multiphoton microscopy (IVI-MP) and image analysis

IVI-MP of calvaria (top of the skull) bone marrow or s.c. tumors in mice were performed within 18-23 days after tumor cell injection. Surgeries were conducted as terminal procedures (VD3390x1) [38, 39, 59, 60]. Animals were intravenously injected with Texas Red-labeled 70 kDa dextran (Thermo Fisher Scientific [38]) 30 min before surgery, mice were anesthetized with continuous isoflurane inhalation. The top of calvaria or tumor tissues were exposed by skin flap surgery and imaged in a custom-designed mouse holder (CHUV, In Vivo Imaging Facility, Lausanne, Switzerland). Imaging was performed on an upright Leica TCS SP8 DIVE multi-photon microscope with an InSight X3 tunable laser (Spectra Physics), two non-descanned hybrid 4Tune detectors [38, 39], and a ×16 multi-immersion objective (HC FLUOTAR L N.A. 0.6 FWD 2.5 mm). For each mouse, multiple z-stacks or a tile scan of the whole bone marrow or tumor tissue was taken with Z-steps of 5–10 μM apart. The representative images of second harmonic generation (SHG), green fluorescent protein (GFP), and Texas Red dextran are shown in pseudo-color; brightness needed to be adjusted in some representative merged images.

### Statistical analysis

Statistical significance comparing groups was determined with the Mann–Whitney test for Figs. 1–3. Data in Fig. 4 was analyzed by two-way analysis of variance, and one-way and two-way analysis of variance was used to analyze results in Figs. 5–7. *p* values < 0.05 were considered statistically significant. Central values represent medians or means, as specified in each figure legend; curves represent means; error bars represent s.e.m.

### Supplementary information

Supplementary Appendix

Movie S1

Movie S2

## Data Availability

All data generated or analyzed during this study are included in this published article and its supplementary information files.
