# Supplementary material for: Macrophage migration inhibitory factor blockade reprograms macrophages and disrupts prosurvival signaling in acute myeloid leukemia
Source: Cell Death Discov. 2024 Mar 28;10:157. doi: 10.1038/s41420-024-01924-5 (PMC10978870; doi:10.1038/s41420-024-01924-5)
Supplement: Supplementary file 1 — Supplementary Appendix [file 41420_2024_1924_MOESM1_ESM.docx]

**Supplementary Appendix:**

Macrophage migration inhibitory factor blockade reprograms macrophages and disrupts prosurvival signaling in acute myeloid leukemia

Caroline Spertini*^1^, Alexandre P. Bénéchet*^2^ , Flora Birch^3, 4^ _,_ Axel Bellotti^1^, Mónica Román-Trufero^1^, Caroline Arber^1,3,4,5,6^_,_ Holger W. Auner^1,5^ , Robert A. Mitchell^7^_,_ Olivier Spertini^5^_,_ and Tatiana Smirnova^1^

*These authors contributed equally

^1^Service and Central Laboratory of Hematology, Centre Hospitalier Universitaire Vaudois, 1011 Lausanne

^2^In Vivo Imaging Facility (IVIF), Department of Research and Training, Lausanne University Hospital and University of Lausanne, Lausanne 1011, Switzerland.

^3^Department of oncology UNIL-CHUV, Centre Hospitalier Universitaire Vaudois, 1011 Lausanne, University of Lausanne (UNIL), Switzerland

^4^Ludwig Institute for Cancer Research Lausanne, 1015 Lausanne, Switzerland

^5^Faculty of Biology and Medicine, University of Lausanne, 1011 Lausanne, Switzerland

^6^Service of immuno-oncology, Centre Hospitalier Universitaire Vaudois, 1011 Lausanne

^7^Department of Surgery, Division of Immunotherapy, University of Louisville, Louisville, KY 40202, USA

**Table of contents**

**Supplementary Materials and Methods**

**Supplemental figures**

**Figure S1.** Primary AML blasts, MΦs, and HD-derived MΦs secrete MIF and express MIF receptors on their cell surface

**Figure S2.** MIF inhibition lowers cell proliferation and the pan-caspase inhibitor Z-VAD-FMK partially rescues leukemia cell lines from MIF inhibitor-induced apoptosis.

**Figure S3.** The MIF inhibitor ISO-1 does not efficiently reprogram M-MΦs alone or in combination with GM-CSF

**Figure S4.** CD80 expression is increased on macrophages after reorientation

**Figure S5.** 4-IPP induces myeloblast apoptosis in co-culture experiments

**Figure S6.** Autoradiographies of cytokine array membranes quantified in Table S2

**Figure S6.** Contact with M-MΦ downregulates Bcl-2 levels in leukemia cells.

**Figure S8.** Leukemia cell engraftment in spleens, and representative skull bone marrow image mosaics from IVI-MP of mice from each treatment group.

**Figure S9.** Representative whole tumor image mosaics from IVI-MP of mice from each treatment group.

**Figure S10.** Autoradiographies of cytokine array membranes quantified in Table S3

**Supplemental tables**

**Table S1.** 2016 WHO classification, ELN 2017 genetic risk, FAB classification and gene mutations from patients included in the study.

**Table S2.** Relative quantification of cytokines from membranes of Figure S6

**Table S3.** Relative quantification of human tumor cytokines from membranes of Figure S10

**Table S4.** Relative quantification of murine tumor cytokines from membranes of FigureS10

Pages 3-5

Page 6

Page 7

Page 8

Page 9

Page 10

Page 11

Page 12

Page 13

Page 14

Page 15

Page 16

Page17

Page18

Page19

**Supplemental Movies** Page 20

Legend **Movie S1.** Animation example of IVI-MP of skull bone marrow

Legend **Movie S2.** Animation of image analysis approach from IVI-MP of a tumor mosaic

**References** Page 21

**Supplementary Materials and Methods.**

**Cell lines**

HL-60 - acute myeloid leukemia with maturation (ATCC#CCL-240), NB4 - acute promyelocytic leukemia (DSMZ#ACC 207), OCI-AML3 - NPM1 mutated (DSMZ#ACC 582), and U937 - monoblastic leukemia (DSMZ#ACC 5) cell lines were cultured in Roswell Park Memorial Institute (RPMI) 1640/10% fetal bovine serum (FBS) = plain medium (PM); MV-4-11 - monoblastic AML with MLL-AF4 and FLT3-ITD (FLT3 with internal tandem duplication) mutations (ATCC#CRL-9591) were cultured in Iscove’s Modified Dulbecco Medium/10% FBS. The endothelial cell line EA.hy926 (ATCC#CRL-2922) and the HS-5 stromal cell line (ATCC#CRL11882) were cultured in Dulbecco's Modified Eagle Medium + 10% FBS. MOLM-13 cells were purchased from DSMZ (ACC#554), and cultured in RPMI 1640 and 20% FBS. All cells were maintained in a humidified atmosphere containing 5% CO_2_ at 37°C, as described previously (1). U937 cells were transduced with retroviral particles encoding for Green Fluorescent Protein (GFP) and firefly luciferase (FFLuc) (GFP-FFLuc) as previously described (2). U937.GFP-FFLuc cells were FACS sorted and maintained at > 98% purity.

**Healthy donor-derived macrophages**

The monocytic fraction was isolated by centrifuging healthy donor (HD) peripheral blood as previously described (1), anticoagulated with citrate phosphate dextrose-adenine 1 and diluted 1/2 with PBS for 10 min at 1000 g to prepare a buffy coat. The concentrated leukocytic fraction was then centrifuged at 400 g for 40 min on a Ficoll-Paque PLUS (GE Healthcare) gradient. Mononuclear cells were collected and distributed in 24-well plates (1.4 x 10^6^ cells/well) for differentiation into macrophages (MΦ) by culturing them for one week in PM supplemented with 10 ng/mL recombinant human M-CSF (ImmunoTools), (M-MΦ), or in AML CM (= conditioned medium), prepared by harvesting, double centrifugation, and filtering medium from cultured patient blasts or above-listed AML cell lines, and diluting it 1:1 with PM (1) (CM-MΦ); medium was changed every other day, as described previously(1). MΦ were detached from the wells in phosphate-buffered saline (PBS) containing 10 mM ethylenediaminetetraacetic acid, and washed with RPMI 1640; the expression of CD163 (333612, Biolegend) (together with CD45 (B36294, Beckman Coulter) and CD14 (R086401-2, Dako)) was verified by flow cytometry (FC) before switching the culture medium to reprogramming conditions for another week using GM-CSF (GM, 1000 U/mL, Miltenyi Biotec), or 4-IPP (IPP, 50 μM, Tocris), or ISO-1 (indicated concentrations, Medchem) alone or in combination. Following this, CD163 expression was verified again by FC. For testing the effect of the inhibition of MIF activity on MΦ polarization, healthy donor-derived monocytes were cultured in AML CM +/- 50 μM 4-IPP for 7 days, before CD163 analysis by FC.

**Patient sample flow cytometry**

After red cell lysis with 0.8% ammonium chloride and cell washes with PBS with FBS, patient samples were prepared for *in vitro* co-culture experiments and FC, performed by multiple parameter analyses using a 10-color/24-antibody panel and a Gallios cytometer (Beckman Coulter), as described (1), according to 2017 European LeukemiaNet recommendations (3). Three additional monoclonal antibodies (mAb) were included in the panel: anti-CD74 (clone LN2), anti-CD44 (clone BJ18), and anti-CD184 (clone 12G5), all from Biolegend.

**AML cell line apoptosis in response to inhibitors and in co-cultures with macrophages**

Blast cell apoptosis was assessed 72 h after incubation with MIF-inhibitors by myeloblast staining with Annexin V and 7-aminoactinomycin D (Beckman Coulter), and multiparameter FC analysis. Z-VAD-FMK used to prevent caspase-dependent apoptosis was from TargetMol. Proliferation of leukemia cell lines was assessed using PKH26 cell linker kit (Sigma–Aldrich) as described previously (1) and measuring the mean fluorescence intensity by FC. In experiments studying the impact of interactions with MΦ on blast survival, MΦ monolayers were washed with PBS before adding myeloblasts (1.5-5 x 10^4^ cells/24-well) suspended in 0.6 mL PM. Blast cell apoptosis was assessed 96 h later by myeloblast staining with Annexin V and 7-aminoactinomycin D (Beckman Coulter), and multiparameter FC analysis. Z-VAD-FMK used to prevent caspase-dependent apoptosis was from TargetMol. MΦ were discriminated from myeloblasts using FS/SS scattergrams, and the anti-CD14, and anti-CD45 mAb.

For testing the impact of interactions with MΦ on leukemia cell line resistance to venetoclax and midostaurin (LC Laboratories), MΦ monolayers were washed with PBS before adding myeloblasts (1.5 x 10^5^ cells/24-well) suspended in 1 mL PM. Blast cell apoptosis was assessed 48 h later by staining with Annexin V and 7-aminoactinomycin D, and FC analysis.

**Immunoblotting**

For testing the impact of interactions with MΦ on BCL-2 levels in leukemia cell lines, HL-60 and MV-4-11 cells were co-cultured as above. After 48h of culture on plastic or macrophage monolayers, HL-60 and MV-4-11 cells were gently collected, spun to remove supernatant, washed, and lysed. Protein samples were separated by SDS-PAGE on a 10% polyacrylamide gel and analyzed by immunoblotting. The mouse anti-Bcl-2 (sc-7382) was purchased from Santa Cruz Biotechnology (Dallas, TX, USA) and the rabbit anti-actin (#4967) was purchased from Cell Signaling Technology (Danvers, MA, USA). After incubation with primary antibodies, the membrane was incubated with goat anti-rabbit IgG conjugated with HRP (#7074, Cell Signaling Technology) and horse anti-mouse IgG conjugated with HRP (#7076, Cell Signaling Technology) and revealed with Clarity Western ECL Substrate (#170-5060, BIO-RAD Laboratories, Hercules, CA, USA). Uncropped blot shown in Figure S6C.

**Blast apoptosis analysis in primary co-cultures**

After red cell lysis with 0.8% ammonium chloride and cell washes with PBS with FBS, all primary cells remaining in patient BM samples were plated as co-cultures in 24-well plates, at a density of 1.5 x 10^6^/well as described previously (1), in PM with or without the studied reagents (DMSO, 10 ng/ml M-CSF, 50 μM 4-IPP, 1000 U/mL GM-CSF). Patient samples processed and plated for assays exhibited spontaneous rates of apoptosis in PM or PM+DMSO < 30% Blast cell death was identified by Annexin V cell staining and multiparameter FC analysis using FS/SS scattergrams and blast staining with the anti-CD45, -CD34, -CD117 mAb (Beckman Coulter) (1). For studying the impact of macrophage orientation on primary blast survival, patient samples with a minimum of 10% MΦ were selected, determined by “day 0” FC analyses before coculture plating. Adherent primary MΦ were detached from the co-culture wells with PBS + 10 mM ethylenediaminetetraacetic acid, for 10 min at 37°C, washed with RPMI 1640 and stained with appropriate mAb for FC analysis.

**Leukemia cell apoptosis in co-cultures with stromal** **cells**

For co-cultures in presence of 4-IPP, 4-6.5 x 10^4^ blasts were added on 3.5 x 10^4^ EA.hy926 or HS-5 that had been plated 24 h before, or on M-MΦs that had been polarized for 1 week in M-CSF and whose level of expression of CD163 had been checked by FC. Wells devoid of adherent cell monolayers (called “plastic”) were used for comparison and controls. Co-cultures were done in presence of DMSO or 50 μM 4-IPP and either in direct contact or separated by 0.4 μm-pore membrane inserts (Transwell, TW, Falcon). After 72 h, myeloblasts were carefully removed for apoptosis analysis by FC with Annexin V (eBioscience) staining, without detaching the adherent cells, which are resistant to 4-IPP.

**Cytokine arrays**

The CM of AML cell lines (1-5 x 10^4^/well), primary blasts (0.5-1 x 10^6^/well), or MΦ monolayers was collected after 3-5 days of culture in 24-well plates. For each membrane, CM from 3 biological repeats were mixed in equal proportions and used for analysis with human XL cytokine array (ARY022B, R&D Systems) according to the manufacturer’s protocol. Quantification was performed with ImageQuant software (Molecular Dynamics), as described (1). Briefly, the mean pixel density of the negative controls was subtracted from the mean of each cytokine duplicate and the mean of each duplicate was then normalized to the mean pixel density of the six reference spots. These normalized values are shown in Table S2, alongside data from M-MΦ previously published (1). Full membranes shown in Figure S5.

For tumor cytokine analyses, U937 tumors were harvested, and pieces of similar weight placed in PM for 24 h; volume of PM was normalized to tissue weight. For cytokine arrays, the CM of 2 individual tumors from each group were pooled and a cytokine array was carried out using the protocol provided by the manufacturer (R&D Systems, ARY028 and ARY022B)(4). Quantification was performed as above. The normalized values are shown in Tables S3 and S4. Full membranes shown in Figure S9.

**Flow cytometry of mouse tissues and macrophages**

For flow cytometry analyses, spleens were dissected out and bone marrow from femurs and tibias were flushed out at the end of experiments from systemic leukemia xenograft mice from the four treatment groups; tissues were mechanically dissociated. Tumor tissues from subcutaneous xenograft mice were also removed and mechanically dissociated. All tissues were passed through a filter. Cell suspensions were incubated with specific antibodies (Biolegend) as described (4). Samples were analyzed for GFP, CD45, and specific markers with a Gallios flow cytometer (Beckman Coulter International S.A.). The reference macrophage population for gating was CD11b and F4/80. From these, inflammatory M1-like MΦ were identified as CD86^+^ cells and protumoral M2-like MΦ as CD206^+^ cells as described previously(4). Anti- CD86 (105024), CD206 (141720), F4/80 (123110), and CD11b (101212), were from Biolegend. Anti-human CD45 (B36294) was from Beckman Coulter, and used as described (1).

### *In vivo* mouse xenograft models: measurements of leukemia progression

U937.GFP-FFLuc cells were systemically engrafted in NSG mice by tail vein injection, and engraftment (5) was confirmed by bioluminescence imaging by day 5 on IVIS Lumina S5 (Revvity), 10-20 min after injection by of D-luciferin (Biosynth AG) 10 mg/ml PBS, 100 ml per mouse. U937.GFP-FFLuc cells were injected s.c. into the right flanks of NSG mice, and engraftment was verified for each mouse by bioluminescence imaging at 24 h. Tumor progression was measured every 2 days by caliper measurement until the end of the experiment, volume was calculated using the formula: Volume = Height × ((Diameter/2)^2^× *π*). All mice were monitored according to the approved score sheet.

**IVI-MP image analysis**

High-resolution three-dimensional images were imported into Imaris version 9.9.1 (Bitplane). MΦ, tumor cells, and blood vessels were classified and segmented using the surface creation tool. Features of individual surfaces were exported as .csv files for each individual z-stack, as performed previously (6, 7).

**Figure S1**


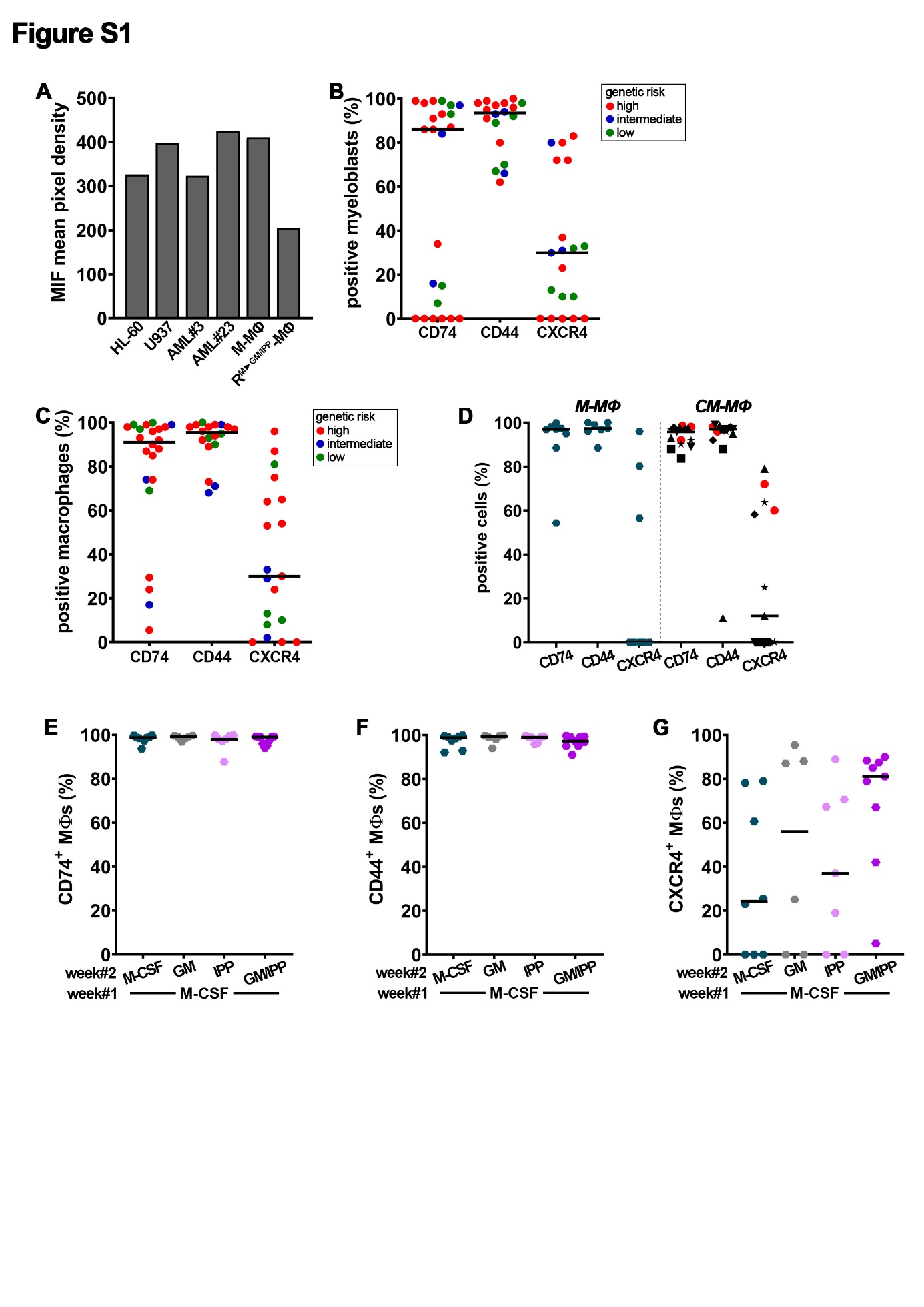


**Figure S1. Primary AML blasts, MΦs, and HD-derived MΦs secrete MIF and express MIF receptors on their cell surface.** (A) Relative quantification, by cytokine array, of MIF contained in pooled culture medium of the indicated leukemia cell lines, primary blasts from patients #3 and #23, and healthy donor-derived macrophages; one membrane was used for relative quantification per pooled CM condition shown. (B,C) Cell surface expression (%) of MIF receptors, CD74, CD44, and CXCR4 on primary myeloblasts (B) and macrophages (C) from bone marrow samples obtained at diagnosis. Horizontal line represents median. Colors indicate patient risk classification. Each dot represents a single patient, n = 18-23. (D) Surface expression (%) of MIF receptors, CD74, CD44, and CXCR4, on healthy donor-derived macrophages cultured for 7 days in presence of M-CSF (left side of the panel, teal hexagons) or conditioned medium (CM) from primary blasts or leukemia cell lines (right side of the panel). Origin of CM: ★ = HL-60, ◼ = MV-4-11, ▲ = NB4, ◆ = U937, ▼ = OCI-AML3, red dots = patients with high genetic risk. The horizontal lines represent the median; n = 7-18. (E,F,G) Surface expression (%) of CD74 (E), CD44 (F), and CXCR4 (G) on healthy donor macrophages after two weeks of culture in culture medium supplemented as indicated. n = 6-9 HD.

**Figure S2**


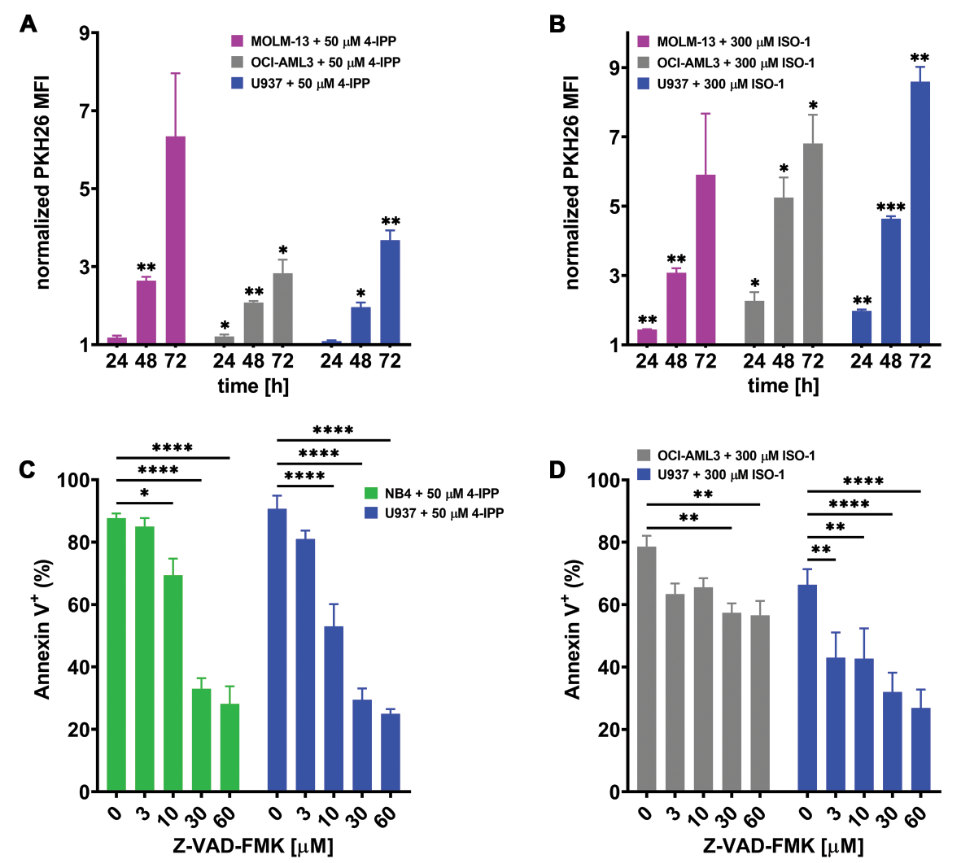


**Figure S2. MIF inhibition lowers cell proliferation and the pan-caspase inhibitor Z-VAD-FMK partially rescues leukemia cell lines from MIF inhibitor-induced apoptosis.** (A, B) MOLM-13, OCI-AML3 and U937 stained with PKH26 (10 μM) were cultured for 24-72 h with 50 μM 4-IPP (A) or 300 μM ISO-1 (B) vs. DMSO and PKH26 mean fluorescence intensity (MFI) of treated cells was normalized to the MFI of cells cultured in DMSO. The mean + SEM of 3 independent experiments is illustrated. * *p* < 0.05, ** *p* < 0.005, *** *p* < 0.001 compared to culture in DMSO. (C) NB4 and U937 were cultured for 72 h with 50 μM 4-IPP and increasing doses of Z-VAD-FMK (0-60 μM), and then stained with Annexin V for analysis of apoptosis by flow cytometry. (D) OCI-AML3 and U937 were cultured for 72 h with 300 μM ISO-1 and increasing doses of Z-VAD-FMK (0-60 μM), and then stained with Annexin V for analysis of apoptosis by flow cytometry. The mean +/- SEM of 4-8 independent experiments is illustrated. * *p* < 0.05, ** *p* < 0.005, **** *p* < 0.0001 compared to viability of cells cultured without Z-VAD-FMK.

**Figure S3**


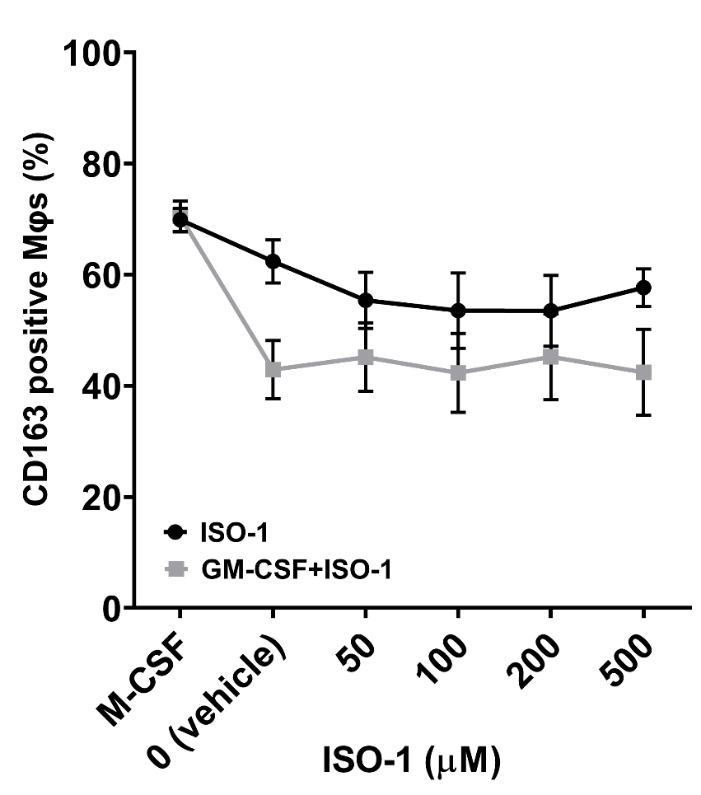


**Figure S3. The MIF inhibitor ISO-1 does not efficiently reprogram M-MΦs alone or in combination with GM-CSF**. M-MΦs from 3 healthy donors were cultured for 7 days in M-CSF, and then 7 more days in the indicated conditions for testing reprogramming. Curves show the % CD163 surface expression on M-MΦs with increasing doses of the ISO-1 tested alone or in combination with GM-CSF.

**Figure S4**


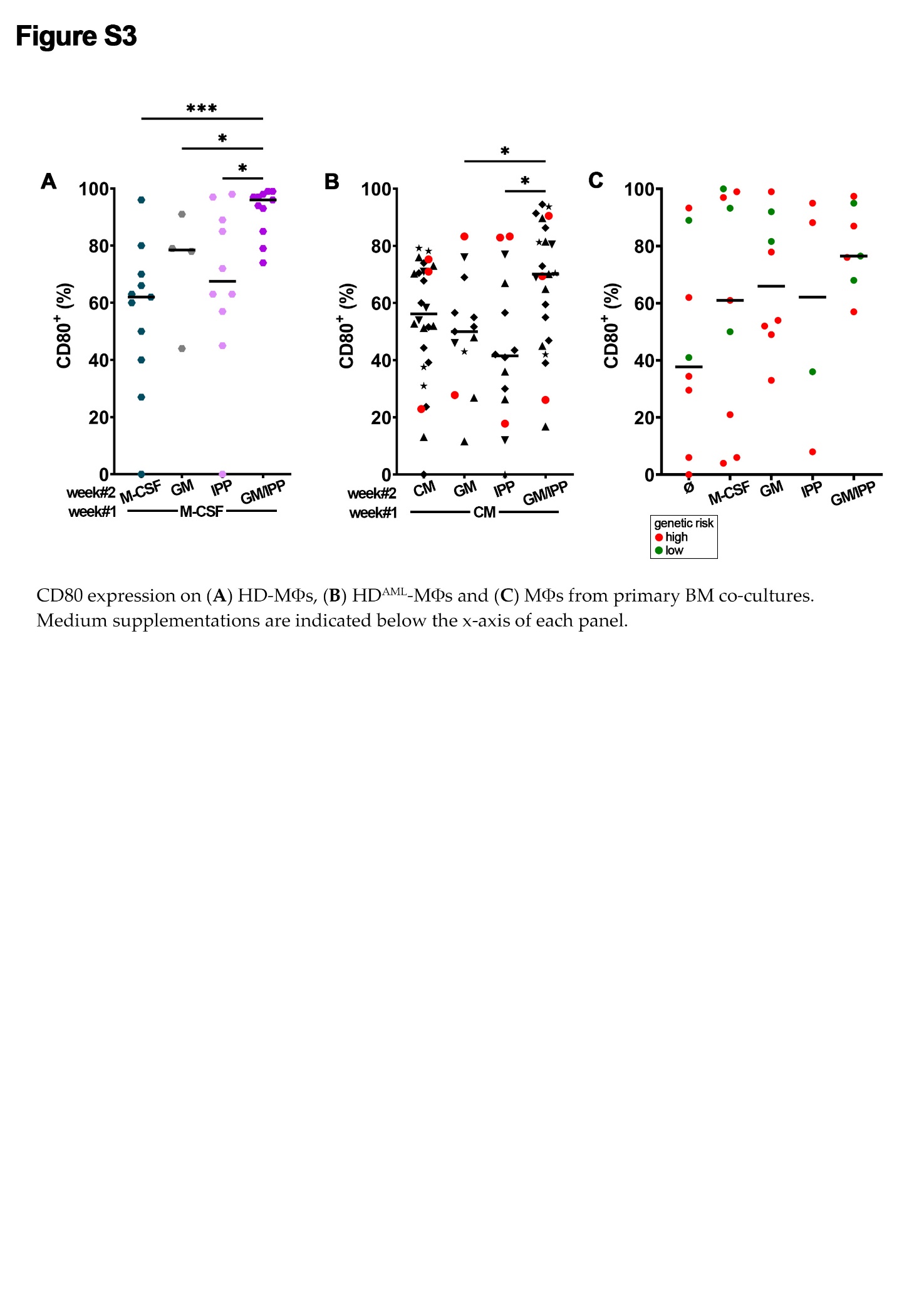


**Figure S4. CD80 expression is increased on macrophages after reorientation**. CD80 expression on (A) M-MΦ, n= 4-11 HD; (B) CM-MΦ, n = 13-26 measurements from 4-9 HD; and (C) MΦ from primary bone marrow co-cultures, n= 4-9 different patient samples. Medium supplementation is indicated below the x-axis of each panel.

**Figure S5**


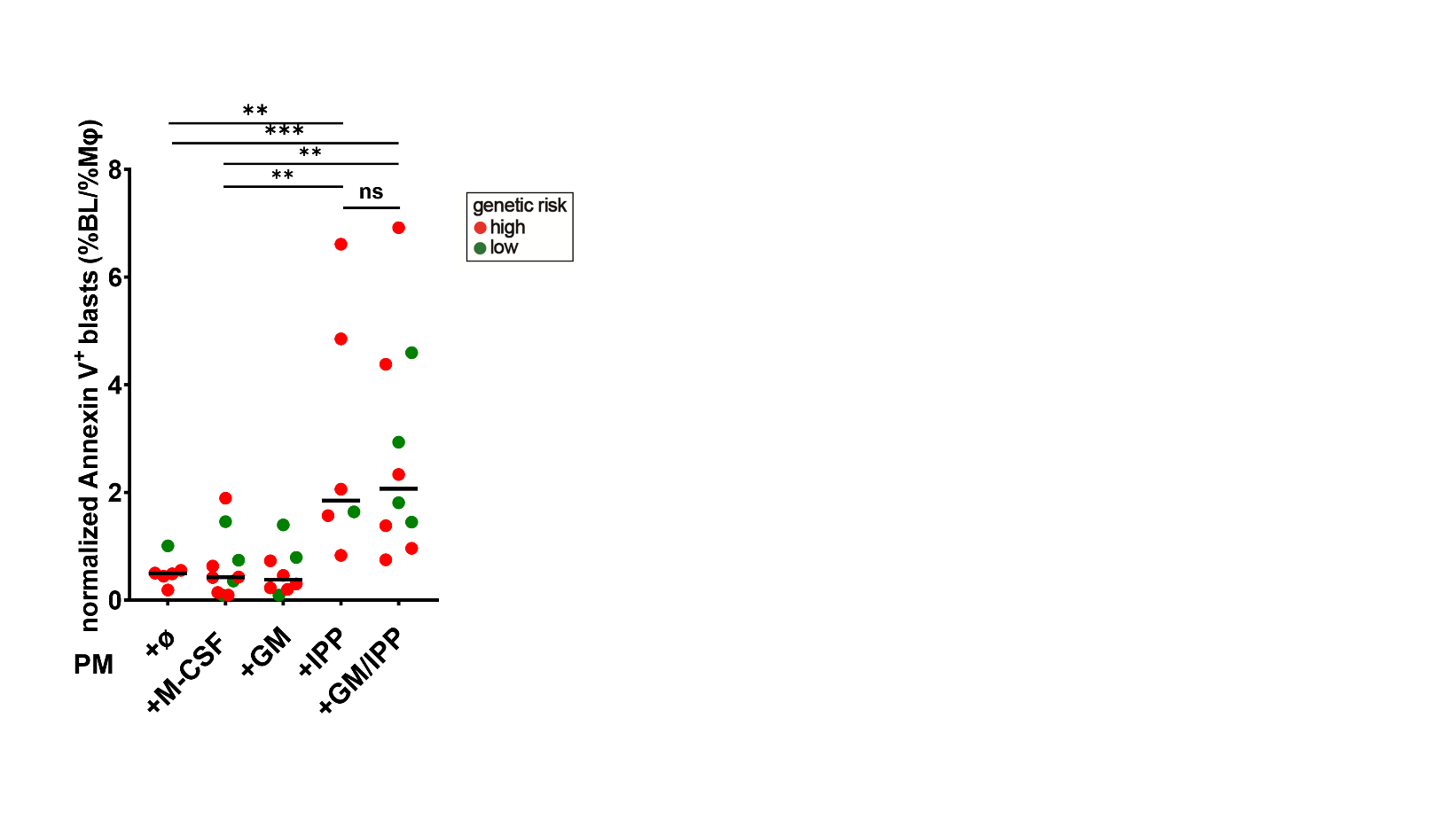


**Figure S5. 4-IPP induces myeloblast apoptosis in co-culture experiments.** Primary myeloblast apoptosis induced after 4-7 days in indicated BM co-culture conditions; % Annexin V+ blasts normalized to % MΦ in each sample; PM = plain medium; n = 6-10. Horizontal lines represent the median apoptosis frequency; ** *p* < 0.005, *** *p* < 0.001.

**Figure S6**


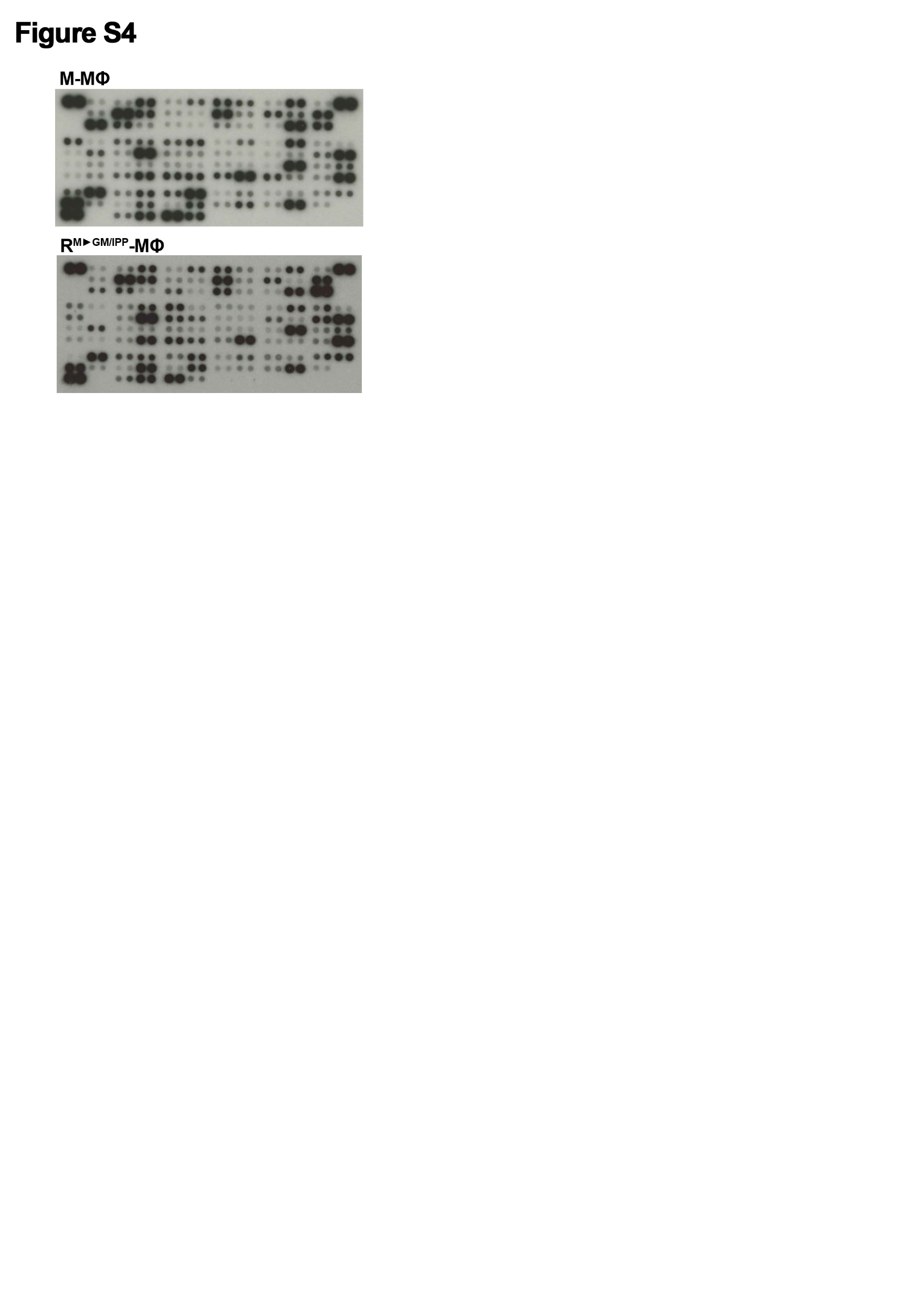


**Figure S6. Autoradiographies of cytokine array membranes quantified in Table S2.** Origin of CM is indicated above each membrane. M-MΦ array analysis has already been published (https://doi.org/10.3390/cancers13215289) and is illustrated here for comparison purpose only.

**Figure S7**


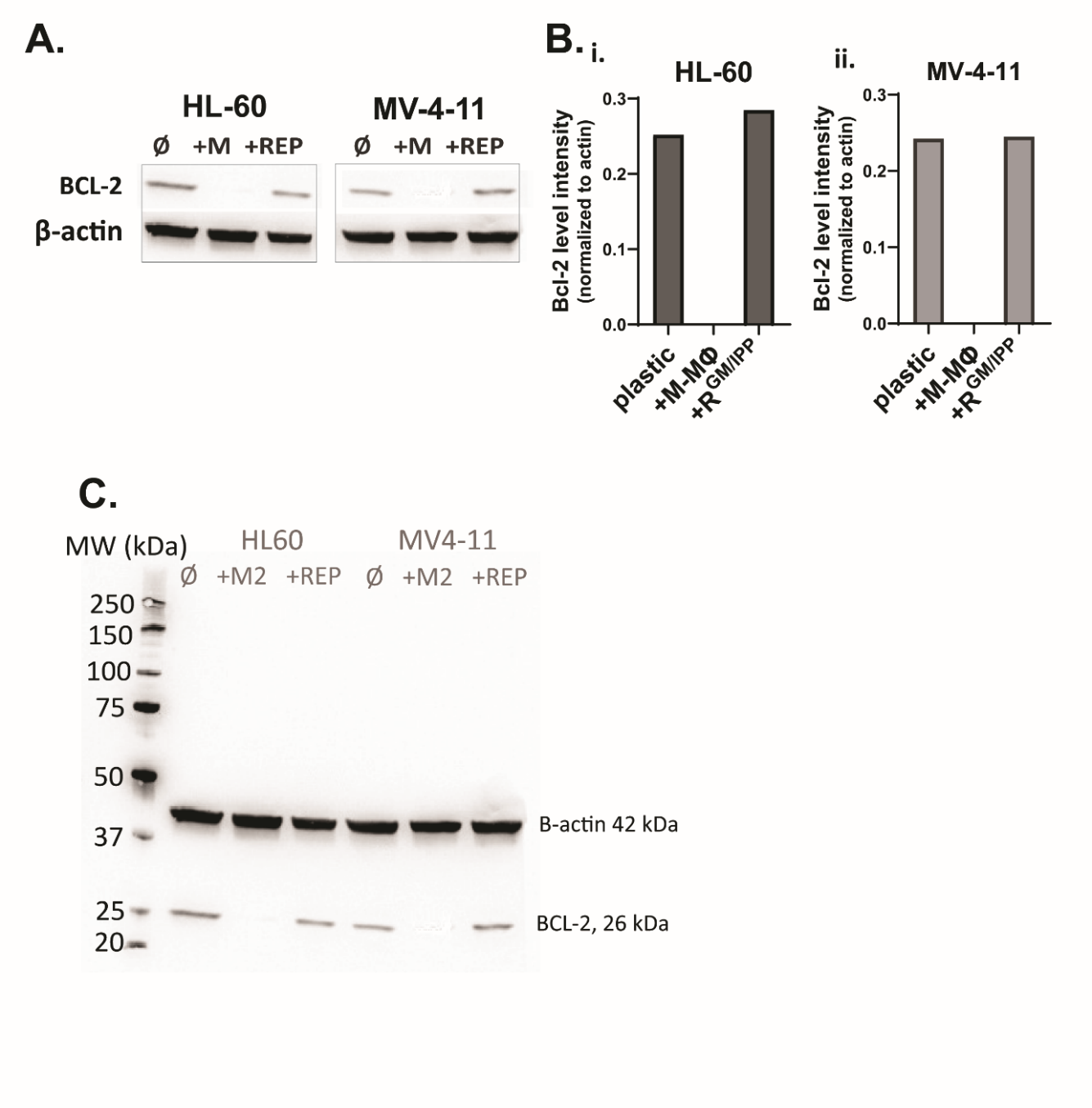


**Figure S7. Contact with M-MΦ downregulates Bcl-2 levels in leukemia cells**. (A) Lysates from HL-60 and MV-4-11 AML cells cultivated for 48 h on “plastic” or with indicated protumoral or reprogrammed MΦ monolayers were immunoblotted with anti-BCL-2 antibody (26 kDa) and anti-β-actin (42 kDa) antibody as loading control. (B) BCL-2 bands from i.) HL-60 cells and ii.) MV-4-11 cells, were quantified and normalized to respective β-actin bands. (C) Uncropped original Western blot.

**Figure S8**


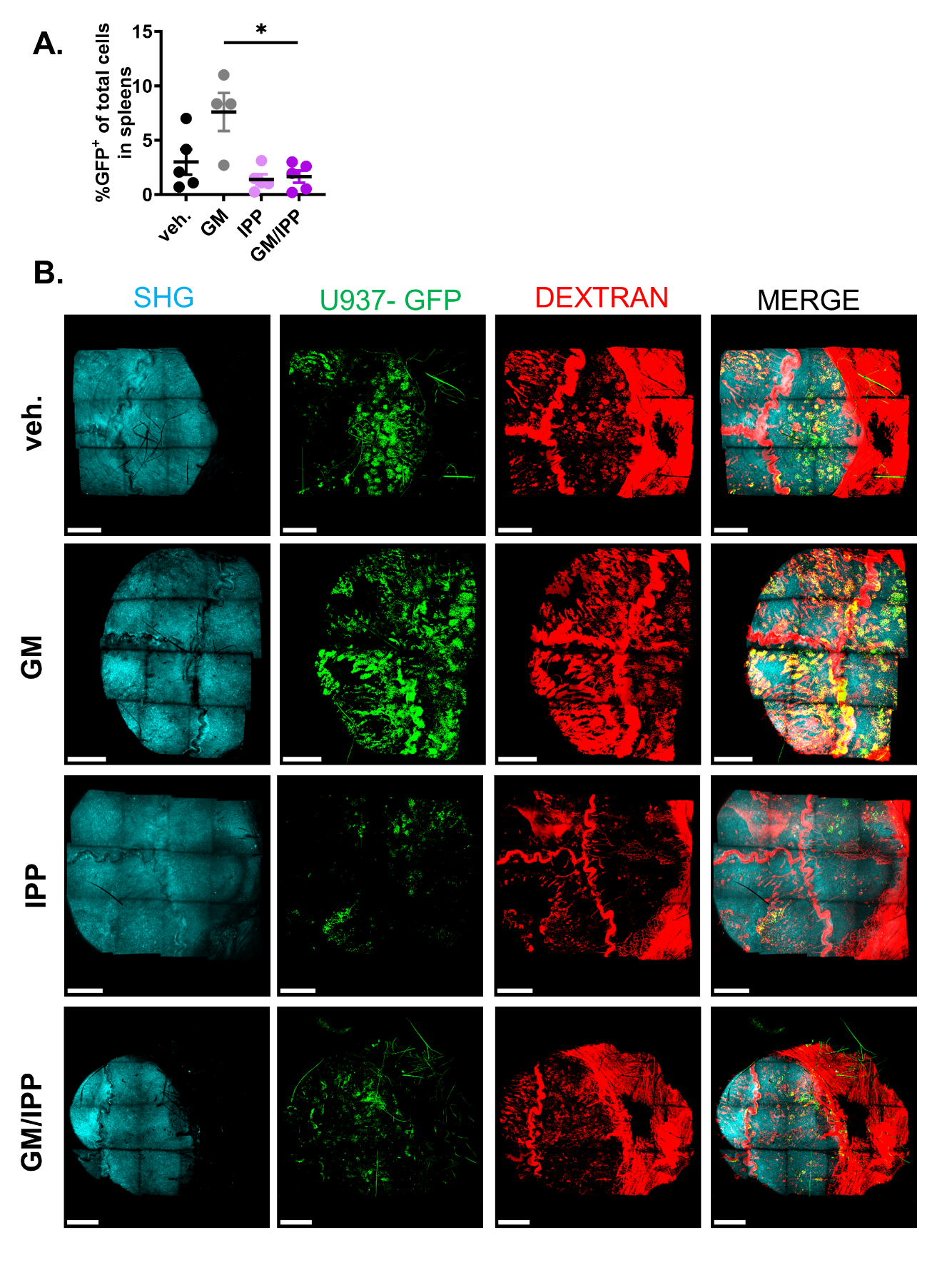


**Figure S8.** A. Flow cytometry analyses or the proportions of humanCD45+ and GFP+ U937 AML cells in spleens of systemically inoculated (i.v.) xenograft NSG mice treated as indicated: veh. = vehicle, GM = GM-CSF, IPP = 4-IPP, GM/IPP = GM-CSF + 4-IPP. B. Representative whole top skull image mosaics from IVI-MP of bone marrow in mice from each treatment group, show each channel acquired during IVI-MP separately, and together (merged): second harmonic generation/SHG imaging identified collagen I fibers – bone structure (cyan), U937-GFP leukemia cells (green), dextran-labeled blood vessels and positive phagocytic cells (red); veh. = vehicle, GM = GM-CSF, IPP = 4-IPP, GM/IPP = GM-CSF + 4-IPP, scale bars = 1000 µm.

**Figure S9**


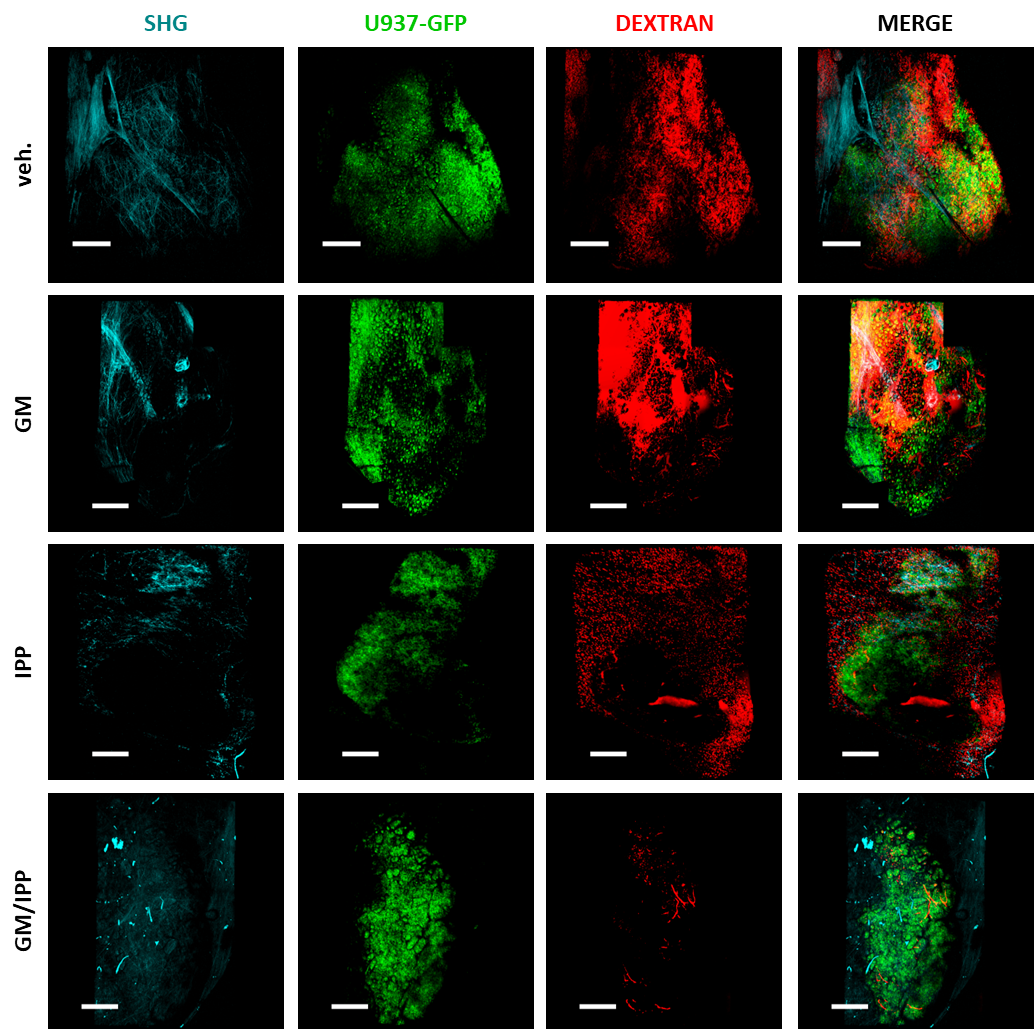


**Figure S9. Representative whole subcutaneous tumor image mosaics from IVI-MP of mice from each treatment group**, show each channel acquired during IVI-MP separately, and together (merged): second harmonic generation/SHG imaging identified collagen I fibers (cyan), U937-GFP leukemia cells (green), dextran-labeled blood vessels and positive phagocytic cells (red); veh. = vehicle, GM = GM-CSF, IPP = 4-IPP, GM/IPP = GM-CSF + 4-IPP, scale bars = 500 µm.

**Figure S10**


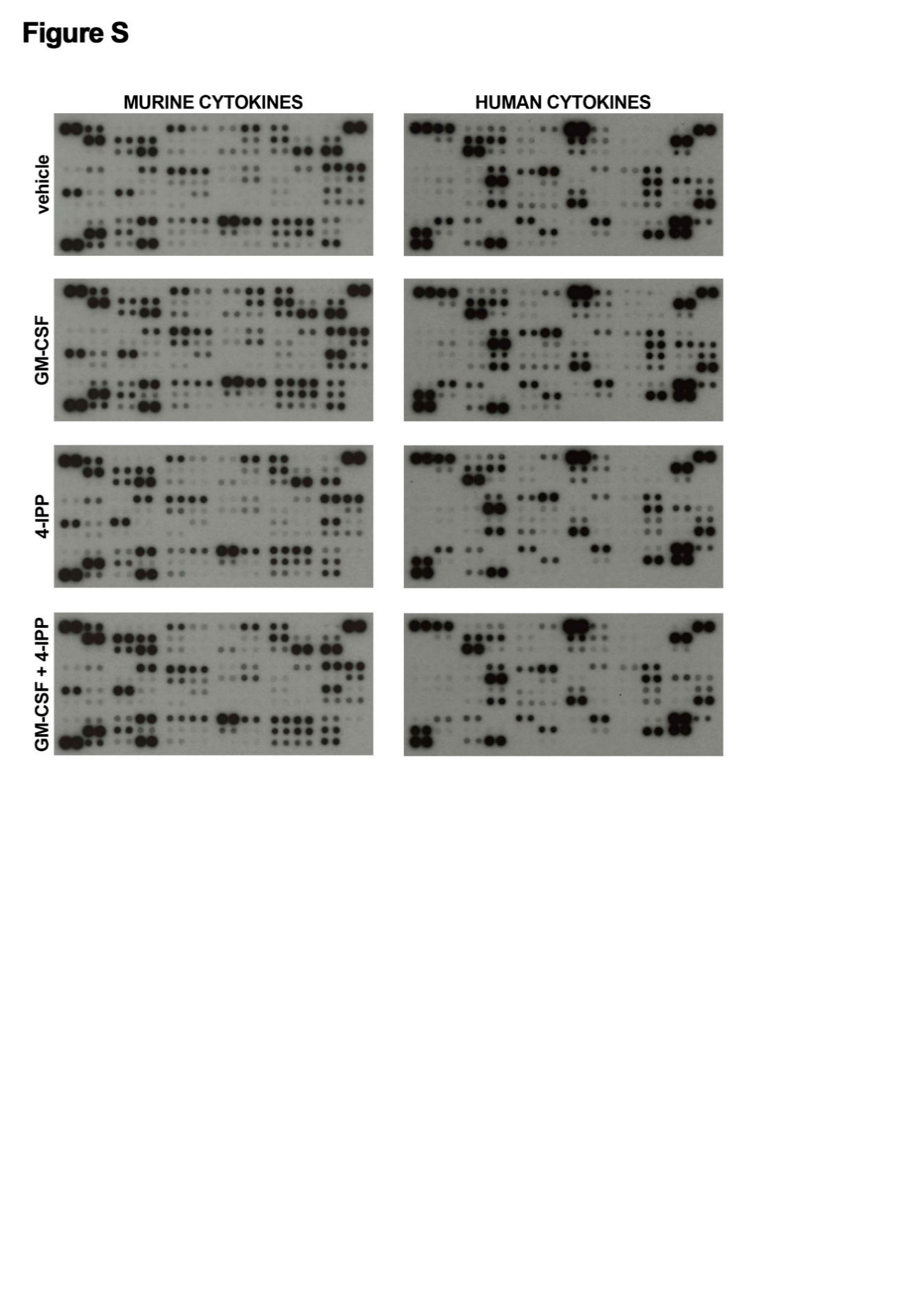


**Figure S10. Autoradiographies of cytokine array membranes quantified in Table S3**. Origin of murine or human tumor CM is indicated to the left of membranes. Quantification of all cytokines are indicated in Tables S3 and S4.

**Table S1. 2016 WHO classification, ELN 2017 genetic risk, FAB classification, and gene mutations from patients included in the study.**

| **patient #** | **2016 WHO classification** | **ELN 2017 genetic risk** | **FAB classification** | **Gene mutations** |
| --- | --- | --- | --- | --- |
| 2 | AML with RGA | Favorable | AML M4 | CBFb-MYH11 |
| 3 | AML with RGA | Favorable | AML M5 | NPM1; TET2; DNMT3A; WT1 |
| 4 | AML with RGA | Favorable | AML M4 | NPM1; NRAS; WT1 |
| 5 | AML with RGA | Favorable | AML M4 | NPM1; DNMT3A; PTPN11; WT1 |
| 6 | AML with RGA | Favorable | AML M2 | NPM1 |
| 7 | AML with RGA | Favorable | AML M2 | NPM1 |
| 9 | AML with RGA | Favorable | AML M2 | NPM1; FLT3-ITD^high^; KIT; TET2 |
| 10 | AML with maturation | Intermediate | AML M2 | DNMT3a; IDH2; NRAS |
| 13 | AML with maturation | Intermediate | AML M2 | FLT3-ITD^high^; NPM1; DNMT3a; WT1 |
| 16 | Monoblastic AML | Intermediate | AML M4 | NPM1; FLT3-ITD^high^ |
| 17 | AML with maturation | Intermediate | AML M2 | NPM1; Flt3-ITD^high^ |
| 19 | AML with MRC | Adverse | AML M5 | KMT2a-MLLT3; EVI1 overexpression |
| 20 | MRC-AML | Adverse | AML M5 | ASXL1; TET2; NPM1; SRSF2; NRAS; KRAS; CBL |
| 22 | Monoblastic AML | Adverse | AML M5 | FLT3-ITD^high^; KMT2A-AFDN |
| 23 | Myelomonocytic AML | Adverse | AML M4 | ASXL1; RUNX1; IDH2; SRSF2 |
| 24 | AML with MRC | Adverse | AML M2 | DNMT3a; IDH1 |
| 25 | AML with maturation | Adverse | AML M2 | STAG2; KMT2A-PTD |
| 27 | AML with MRC | Adverse | AML M4 | ASXL1; EZH2; PTPN11; TET2 |
| 28 | AML with MRC | Adverse | AML M1 | IDH2 |
| 29 | AML with MRC | Adverse | AML M4 | CEBPA ma; TP53 |
| 30 | AML with MRC | Adverse | AML M5 | DNMT3A; ASXL1; RUNX1; SETBP1; SRSF2 |
| 31 | AML with MRC | Adverse | AML M1 | ASXL1; PTPN11; TP53; U2AF1; EVI1 overexpression |
| 34 | AML with MRC | Adverse | AML M4 | ASXL1; KRAS; TET2 |
| 36 | AML with MRC | Adverse | AML M4 | RUNX1; SRSF2; DNMT3A; KMT2A-PTD |
| 37 | Myelomonocytic AML | Adverse | AML M4 | NRAS; RUNX1-USP42 |
| 38 | t-AML | Adverse | AML M5 | KMT2A-MLLT3; WT1; EVI1 overexpression |
| 39 | AML with MRC | Adverse | AML M2 | MLL-PTD; FLT3-ITD^low^; FLT3; DNMT3A; IDH2; WT1 |
| 40 | t-AML | Adverse | AML M4 | IDH2; NRAS; SRSF2; STAG2 |
| 41 | AML with MRC | Adverse | AML M2 | U2AF1; KMT2A-PTD |

MRC: myelodysplasia‐related changes, RGA: recurrent genetic abnormalities, t‐AML: therapy‐related AML, ma: monoallelic.

**Table S2. Relative quantification of cytokines from membranes of Figure S5.**

|  | **M-MΦ** | **R^M⯈GM/IPP^-MΦ** |  |  | **M-MΦ** | **R^M⯈GM/IPP^-MΦ** |  |  | **M-MΦ** | **R^M⯈GM/IPP^-MΦ** |
| --- | --- | --- | --- | --- | --- | --- | --- | --- | --- | --- |
| Adiponectin | 126.2 | 94.9 |  | FasL | 140.5 | 128.5 |  | IL-34 | ND | 77.3 |
| Ang-1 | 83.0 | 83.8 |  | FGF-19 | 176.3 | 599.8 |  | IL-4 | ND | 228.7 |
| Ang-2 | 271.0 | 346.5 |  | FGF-2 | 58.5 | 279.1 |  | IL-5 | 302.3 | 8.8 |
| Angiogenin | 714.7 | 573.2 |  | FGF-7 | ND | 62.4 |  | IL-6 | 114.8 | 142.0 |
| ApoA1 | 151.2 | 193.3 |  | Flt-3 L | 60.9 | 104.1 |  | IL-8 | 958.6 | 1001.8 |
| BAFF | 536.2 | 501.9 |  | G-CSF | ND | 74.5 |  | Kallikrein 3 | 345.2 | 370.4 |
| Basigin | 737.3 | 819.0 |  | GDF-15 | 933.7 | 780.7 |  | Leptin | ND | 91.1 |
| BDNF | 312.2 | 167.0 |  | GH | ND | 49.8 |  | LIF | 137.6 | 42.0 |
| C5/C5a | 70.8 | 114.2 |  | GM-CSF | 696.1 | 1031.4 |  | Lipocalin-2 | 218.7 | 122.9 |
| CCL17 | 380.2 | 859.8 |  | HGF | 235.2 | 137.9 |  | M-CSF | 508.0 | 337.2 |
| CCL19 | 141.5 | 197.6 |  | ICAM-1 | 288.5 | 510.5 |  | MIF | 410.1 | 183.1 |
| CCL2 | 701.1 | 793.7 |  | IFN-γ | 277.1 | 580.9 |  | MMP-9 | 887.5 | 1013.6 |
| CCL20 | 200.3 | 126.1 |  | IGFBP-2 | 448.1 | 79.0 |  | MPO | 320.3 | 36.2 |
| CCL3/4 | 420.9 | 91.5 |  | IGFBP-3 | ND | 111.8 |  | Osteopontin | 929.8 | 806.4 |
| CCL5 | 170.8 | 262.3 |  | IL-1 R4 | ND | 82.6 |  | PDGF-AA | 154.8 | 287.9 |
| CCL7 | 520.9 | 571.8 |  | IL-10 | 145.8 | 451.7 |  | PDGF-AB/BB | 455.7 | 425.6 |
| CD14 | 683.0 | 473.6 |  | IL-11 | 133.5 | 283.5 |  | PECAM-1 | 762.4 | 750.8 |
| CD26 | 459.7 | 396.0 |  | IL-12 p70 | 64.1 | 59.0 |  | PTX3 | 353.5 | 211.5 |
| CD30 | 110.0 | 139.9 |  | Il-13 | ND | 51.0 |  | RAGE | ND | 94.0 |
| CD40L | 176.7 | 119.1 |  | IL-15 | ND | 234.4 |  | RBP-4 | ND | 181.5 |
| CD71 | 487.4 | 603.8 |  | IL-16 | 133.6 | 114.1 |  | Relaxin-2 | 112.1 | 146.6 |
| CFD | 666.7 | 739.1 |  | IL-17A | 244.5 | 617.9 |  | Resistin | 146.2 | 340.5 |
| CHI3L1 | 975.8 | 945.8 |  | IL-18 Bpa | 886.2 | 953.9 |  | Serpin E1 | 1019.9 | 845.3 |
| Cripto-1 | ND | 101.3 |  | Il-19 | ND | 61.3 |  | SHBG | 195.6 | 131.9 |
| CRP | 86.5 | 130.4 |  | IL-1α | 206.2 | 89.4 |  | TFF3 | ND | 112.4 |
| CXCL1 | 427.6 | 204.7 |  | IL-1β | ND | 77.3 |  | TGF-α | 122.8 | 79.3 |
| CXCL10 | 920.3 | 936.1 |  | IL-1ra | 696.2 | 515.1 |  | Thrombospondin-1 | 425.1 | 132.6 |
| CXCL11 | 134.6 | 189.1 |  | IL-2 | 92.7 | 377.5 |  | TIM-3 | 928.5 | 838.9 |
| CXCL12 | 251.7 | 594.0 |  | IL-22 | 102.3 | 348.3 |  | TNF-α | 110.7 | 167.6 |
| CXCL4 | 914.4 | 543.1 |  | IL-23 | ND | 77.2 |  | uPAR | 847.7 | 812.9 |
| CXCL5 | 944.8 | 265.3 |  | IL-24 | 136.1 | 170.0 |  | VCAM-1 | 690.3 | 192.7 |
| CXCL9 | 906.2 | 848.9 |  | IL-27 | 104.2 | 180.8 |  | VEGF | 78.1 | 89.0 |
| Cystatin C | 839.8 | 849.3 |  | IL-3 | ND | 124.9 |  | Vitamin D BP | 208.0 | 238.2 |
| Dkk-1 | 158.7 | 136.6 |  | IL-31 | ND | 94.8 |  |  |  |  |
| EGF | 303.7 | 75.7 |  | IL-32 | 62.1 | 90.0 |  |  |  |  |
| Endoglin | 561.7 | 396.1 |  | IL-33 | ND | 73.0 |  |  |  |  |

The indicated relative optical density of the cytokines are colored from blue (low) to grey (medium) to red (high) according to their mean pixel intensity (ND = not detected). Data of M-MΦ cytokines have already been published (https://doi.org/10.3390/cancers13215289) and are indicated here for comparison purpose only.

**Table S3. Relative quantification of human tumor cytokines from membranes of Figure S9.**

|  | **veh.** | **GM** | **IPP** | **GM/IPP** |  |  | **veh.** | **GM** | **IPP** | **GM/IPP** |
| --- | --- | --- | --- | --- | --- | --- | --- | --- | --- | --- |
| Adiponectin | 844.1 | 835.4 | 842.3 | 806.3 |  | M-CSF | 44.2 | 88.6 | 16.5 | 40.3 |
| Ang-2 | 106.5 | 116.6 | 111.6 | 79.2 |  | MIF | 782.9 | 850.3 | 757.4 | 809.1 |
| Angiogenin | 116.8 | 107.5 | 125.4 | 65.5 |  | MMP-9 | 938.8 | 899.3 | 749.5 | 729.8 |
| ApoA1 | 54.4 | 69.3 | 40.6 | 22.5 |  | Osteopontin | 448.2 | 393.5 | 246.5 | 123.8 |
| BAFF | 1156.4 | 1160.9 | 1117.2 | 1183.1 |  | PAI-1 | 912.8 | 928.2 | 813.6 | 852.8 |
| Basigin | 1035.0 | 1004.8 | 978.5 | 1021.6 |  | PDGF-AA | 152.5 | 165.5 | 100.2 | 151.9 |
| BDNF | 111.6 | 175.6 | 115.5 | 118.1 |  | PECAM-1 | 973.9 | 971.3 | 836.4 | 869.7 |
| CCL2 | 573.9 | 598.6 | 612.0 | 455.7 |  | PTX3 | 423.8 | 447.3 | 240.9 | 300.9 |
| CCL20 | 123.8 | 138.5 | 78.6 | 93.4 |  | Relaxin-2 | 49.0 | 48.9 | 26.7 | 29.6 |
| CCL5 | 579.9 | 571.3 | 508.1 | 536.0 |  | Resistin | 1099.1 | 1096.4 | 1029.6 | 1107.0 |
| CCL7 | 89.5 | 88.6 | 101.7 | 71.0 |  | SHBG | 62.4 | 63.8 | 19.1 | 27.7 |
| CD40L | 94.6 | 79.2 | 67.7 | 36.7 |  | Thrombospondin-1 | 44.7 | 64.4 | 24.5 | 41.1 |
| CD71 | 356.6 | 404.7 | 188.4 | 305.6 |  | TIM-3 | 22.9 | 33.9 | 5.1 | 11.1 |
| CFD | 503.1 | 511.9 | 455.7 | 369.6 |  | uPAR | 788.1 | 821.2 | 722.9 | 766.4 |
| CHI3L1 | 576.4 | 598.7 | 296.6 | 485.1 |  | VCAM-1 | 28.5 | 47.0 | 2.9 | 14.2 |
| CRP | 42.2 | 31.2 | 13.9 | 20.2 |  | VEGF | 1010.3 | 1030.0 | 957.7 | 1005.0 |
| CXCL10 | 342.8 | 351.1 | 84.3 | 104.3 |  | Vitamin D BP | 124.5 | 130.0 | 107.3 | 111.3 |
| CXCL12 | 182.0 | 219.8 | 152.7 | 165.1 |  |  |  |  |  |  |
| Cystatin C | 555.3 | 366.3 | 228.6 | 435.4 |  |  |  |  |  |  |
| Dkk-1 | 84.9 | 108.1 | 91.2 | 96.8 |  |  |  |  |  |  |
| Endoglin | 983.8 | 1006.1 | 943.3 | 996.4 |  |  |  |  |  |  |
| FasL8 | 82.2 | 67.6 | 29.4 | 30.0 |  |  |  |  |  |  |
| FGF-19 | 145.3 | 93.5 | 109.4 | 98.4 |  |  |  |  |  |  |
| GM-CSF | 137.9 | 72.0 | 58.8 | 40.3 |  |  |  |  |  |  |
| ICAM-1 | 340.0 | 404.4 | 183.7 | 260.6 |  |  |  |  |  |  |
| IFN-γ | 215.7 | 286.0 | 164.0 | 202.1 |  |  |  |  |  |  |
| IGFBP-2 | 816.9 | 824.7 | 706.9 | 700.0 |  |  |  |  |  |  |
| IL-11 | 54.9 | 72.0 | 32.7 | 45.7 |  |  |  |  |  |  |
| IL-16 | 586.2 | 630.3 | 489.8 | 529.1 |  |  |  |  |  |  |
| IL-17A | 273.4 | 327.7 | 201.4 | 175.8 |  |  |  |  |  |  |
| IL-18 Bpa | 134.9 | 214.6 | 39.9 | 68.2 |  |  |  |  |  |  |
| IL-1α | 98.1 | 134.7 | 112.3 | 106.4 |  |  |  |  |  |  |
| IL-1β | 52.8 | 64.6 | 10.2 | 54.6 |  |  |  |  |  |  |
| IL-1ra | 323.7 | 314.1 | 274.3 | 313.8 |  |  |  |  |  |  |
| IL-24 | 105.2 | 270.7 | 35.1 | 65.0 |  |  |  |  |  |  |
| IL-32 | 175.8 | 228.7 | 97.4 | 120.3 |  |  |  |  |  |  |
| IL-5 | 38.8 | 62.4 | 13.5 | 20.7 |  |  |  |  |  |  |
| IL-6 | 27.6 | 47.8 | 22.7 | 23.3 |  |  |  |  |  |  |
| IL-8 | 1048.0 | 1050.2 | 1003.7 | 1012.4 |  |  |  |  |  |  |
| Kallikrein 3 | 216.7 | 235.5 | 170.0 | 129.3 |  |  |  |  |  |  |
| Lipocalin-2 | 37.7 | 52.1 | 15.9 | 24.5 |  |  |  |  |  |  |

Mouse treatments are indicated as column titles and the relative optical density of the cytokines are colored from blue (low) to grey (medium) to red (high) according to their mean pixel intensity.

**Table S4. Relative quantification of murine tumor cytokines from membranes of Figure S9.**

|  | **veh.** | **GM** | **IPP** | **GM/IPP** |  |  | **veh.** | **GM** | **IPP** | **GM/IPP** |
| --- | --- | --- | --- | --- | --- | --- | --- | --- | --- | --- |
| Adiponectin | 491.2 | 519.9 | 533.1 | 546.1 |  | IL-1α | 33.8 | 47.7 | 6.8 | 39.0 |
| Ang-2 | 508.4 | 540.4 | 358.8 | 260.5 |  | IL-1ra | 96.8 | 145.6 | 41.4 | 68.8 |
| Angiopoietin-like 3 | 96.9 | 107.6 | 51.2 | 46.5 |  | IL-33 | 127.3 | 241.3 | 115.5 | 114.2 |
| BAFF | 73.5 | 115.9 | 35.3 | 30.7 |  | IL-4 | 35.6 | 59.9 | 53.3 | 30.3 |
| CCL11 | 343.0 | 279.5 | 303.8 | 766.1 |  | IL-6 | 221.5 | 814.8 | 495.8 | 625.4 |
| CCL12 | 478.9 | 455.0 | 449.7 | 601.0 |  | LDL R | 167.9 | 275.6 | 99.2 | 118.7 |
| CCL17 | 16.3 | 43.4 | 16.1 | 34.4 |  | LIF | 96.2 | 161.5 | 62.5 | 105.8 |
| CCL2 | 235.3 | 383.3 | 515.8 | 321.1 |  | Lipocalin-2 | 128.9 | 150.9 | 90.5 | 112.3 |
| CCL21 | 141.1 | 330.9 | 67.4 | 29.5 |  | M-CSF | 163.1 | 244.3 | 107.1 | 209.7 |
| CCL22 | 346.8 | 694.2 | 485.6 | 551.0 |  | MMP-2 | 282.5 | 266.4 | 200.7 | 257.9 |
| CCL6 | 887.8 | 930.0 | 810.5 | 975.4 |  | MMP-3 | 923.6 | 928.5 | 905.3 | 924.7 |
| CD14 | 52.7 | 83.6 | 62.5 | 67.7 |  | MMP-9 | 603.7 | 731.1 | 312.8 | 284.8 |
| CD26 | 114.8 | 139.5 | 55.5 | 90.0 |  | Myeloperoxidase | 411.3 | 570.8 | 494.8 | 496.5 |
| CD40 | 213.1 | 264.5 | 60.9 | 143.6 |  | Osteopontin | 515.9 | 666.4 | 604.5 | 590.2 |
| CD93 | 468.1 | 445.7 | 486.5 | 376.0 |  | Osteoprotegerin | 141.7 | 262.6 | 80.9 | 150.5 |
| Chemerin | 167.4 | 230.6 | 277.8 | 341.8 |  | PAI-1 | 887.9 | 924.2 | 893.0 | 931.5 |
| Chitinase 3-like 1 | 810.4 | 816.6 | 846.5 | 817.8 |  | Pentraxin 2 | 922.4 | 946.2 | 926.1 | 965.2 |
| Complement Factor D | 95.6 | 116.7 | 63.0 | 95.7 |  | Pentraxin 3 | 246.1 | 338.6 | 195.2 | 380.8 |
| CRP | 86.7 | 67.0 | 11.6 | 24.0 |  | Periostin | 42.6 | 122.3 | 47.7 | 118.3 |
| CXCL1 | 672.4 | 788.3 | 825.1 | 849.9 |  | Pref-1 | 43.4 | 53.9 | 16.1 | 19.7 |
| CXCL10 | 134.0 | 32.1 | 166.5 | 93.6 |  | Proprotein Convertase 9 | 180.2 | 178.1 | 114.4 | 137.7 |
| CXCL13 | 289.9 | 264.3 | 458.0 | 598.0 |  | P-selectin | 83.6 | 97.2 | 35.6 | 61.2 |
| CXCL16 | 532.8 | 696.6 | 387.5 | 583.4 |  | RAGE | 28.2 | 35.9 | 13.1 | 12.7 |
| CXCL2 | 878.5 | 917.6 | 520.4 | 883.1 |  | RBP4 | 244.8 | 364.4 | 311.6 | 296.8 |
| CXCL5 | 696.4 | 779.8 | 714.5 | 797.5 |  | Reg3G | 400.5 | 540.4 | 202.5 | 461.3 |
| Cystatin C | 406.4 | 416.9 | 403.1 | 396.1 |  | Resistin | 61.8 | 265.3 | 228.2 | 381.2 |
| Endoglin | 135.8 | 183.6 | 62.1 | 106.7 |  | Serpin F1 | 34.0 | 109.9 | 78.5 | 45.0 |
| Endostatin | 627.1 | 681.1 | 685.3 | 660.7 |  | TF | 59.1 | 135.4 | 54.2 | 59.1 |
| E-selectin | 307.7 | 390.4 | 183.3 | 351.7 |  | VCAM-1 | 98.4 | 183.1 | 117.2 | 160.2 |
| Fetuin A | 686.4 | 692.3 | 667.9 | 670.6 |  | VEGF | 31.7 | 101.3 | 33.0 | 155.6 |
| Fractalkine | 134.7 | 297.4 | 138.2 | 116.4 |  | WISP-1 | 492.3 | 492.1 | 296.4 | 431.1 |
| G-CSF | 71.4 | 300.3 | 141.9 | 186.3 |  |  |  |  |  |  |
| GDF-15 | 47.4 | 67.5 | 31.4 | 78.0 |  |  |  |  |  |  |
| GM-CSF | 16.3 | 36.5 | 26.8 | 48.8 |  |  |  |  |  |  |
| HGF | 153.4 | 211.5 | 82.6 | 194.9 |  |  |  |  |  |  |
| ICAM-1 | 27.3 | 62.8 | 21.4 | 20.6 |  |  |  |  |  |  |
| IGFBP-2 | 249.7 | 332.7 | 114.1 | 230.8 |  |  |  |  |  |  |
| IGFBP-3 | 610.2 | 719.9 | 567.1 | 571.9 |  |  |  |  |  |  |
| IGFBP-5 | 40.9 | 133.2 | 55.9 | 48.5 |  |  |  |  |  |  |
| IGFBP-6 | 415.7 | 665.5 | 608.5 | 798.1 |  |  |  |  |  |  |
| IL-11 | 19.8 | 51.7 | 32.8 | 33.7 |  |  |  |  |  |  |

Mouse treatments are indicated as column title and the relative optical density of the cytokines are colored from blue (low) to grey (medium) to red (high) according to their mean pixel intensity.

**Movie S1. Representative animation example of IVI-MP of a skull bone marrow from a GM-CSF treated mouse.**

Video shows a whole top skull 3-D mosaic (GFP = green, U937 cells, dextran = red, blood vessels, dextran+ cells, and second harmonic generation = blue, collagen I/bone structure); scale bars shown = 400 - 1000 µm.

**Movie S2. Animation of image analysis approach from IVI-MP of a subcutaneous tumor mosaic from a GM-CSF + 4-IPP treated mouse.**

Video shows a whole 3-D tumor mosaic (GFP = green, dextran = red, and second harmonic generation = blue), followed by 3-D surface rendering in the red/dextran channel only (red), then size classification, and the separation of tumor blood vessels (blue) and macrophages (purple); scale bar = 500 µm.

**References**

1. Smirnova T, Spertini C, Spertini O. CSF1R Inhibition Combined with GM-CSF Reprograms Macrophages and Disrupts Protumoral Interplays with AML Cells. Cancers. 2021;13(21).

2. Camviel N, Wolf B, Croce G, Gfeller D, Zoete V, Arber C. Both APRIL and antibody-fragment-based CAR T cells for myeloma induce BCMA downmodulation by trogocytosis and internalization. J Immunother Cancer. 2022;10(11).

3. Béné MC, Nebe T, Bettelheim P, Buldini B, Bumbea H, Kern W, et al. Immunophenotyping of acute leukemia and lymphoproliferative disorders: a consensus proposal of the European LeukemiaNet Work Package 10. Leukemia. 2011;25(4):567-74.

4. Smirnova T, Bonapace L, MacDonald G, Kondo S, Wyckoff J, Ebersbach H, et al. Serpin E2 promotes breast cancer metastasis by remodeling the tumor matrix and polarizing tumor associated macrophages. Oncotarget. 2016;7(50):82289-304.

5. Harris P, Ralph P. Human leukemic models of myelomonocytic development: a review of the HL-60 and U937 cell lines. J Leukoc Biol. 1985;37(4):407-22.

6. Benechet AP, Menon M, Xu D, Samji T, Maher L, Murooka TT, et al. T cell-intrinsic S1PR1 regulates endogenous effector T-cell egress dynamics from lymph nodes during infection. Proc Natl Acad Sci U S A. 2016;113(8):2182-7.

7. Benechet AP, Ganzer L, Iannacone M. Intravital Microscopy Analysis of Hepatic T Cell Dynamics. Methods Mol Biol. 2017;1514:49-61.
